# Novel Gold(I) and Silver(I) Complexes of Phosphorus-1,1,-dithiolates and Molecular Structure of [O,O’-(Bornyl)_2_PS_2_]H_3_NC(CH_3_)_3_

**DOI:** 10.1186/1752-153X-7-89

**Published:** 2013-05-20

**Authors:** Samet Solak, Cemal Aydemir, Mehmet Karakus, Peter Lönnecke

**Affiliations:** 1 Department of Chemistry, Faculty of Arts & Sciences, Pamukkale University, Kinikli Denizli, 20075, Turkey; 2 Institut für Anorganische Chemie, Universität Leipzig, Johannisallee 29, Leipzig D-04103, Germany

**Keywords:** Phosphorus-1,1-dithiolate, Chiral, Silver(I) and gold(I) complexes

## Abstract

**Background:**

The novel chiral phosphorus-1,1-dithiolates [4-CH_3_OC_6_H_4_P(S)(OR)S]^-^[H_3_NC(CH_3_)_3_]^+^ were synthesized by the reaction of [RPS_2_)]_2_ (R = 4-MeOC_6_H_4_) or P_2_S_5_ and the respective alcohol ROH (R = myrtanyl, 2-naphthylethyl, myrtenyl, borneol) in toluene. The reaction of phosphorus-1,1-dithiolates **1**–**4** and Au(tht)Cl, AuClPPh_3_ or AgNO_3_ and PPh_3_ gave rise to gold(I)- and silver(I)-complexes in THF. All compounds have been characterised by elemental analyses, IR, NMR (^1^H-, ^13^C- and ^31^P-) spectroscopy as well as MS measurements. Optical rotation values confirmed the chirality of the compounds. The Compound **4** has been characterized structurally by X-ray crystallography.

**Results:**

Phosphorus-1,1,-dithiolate compounds were formed as liquids and were treated with suitable amine in order to convert them to their salts **1**–**4** . They have been successfully characterized spectroscopically (IR, ^1^H, ^13^C, ^31^P NMR) as well as mass spectra. The compound **4** has been also structurally by X-ray crystallography. The compound **4** crystallizes in the orthorhombic space group P2(1)2(1)2(1) with Z = 4. Compounds containing phosphorus and sulfur donor atoms are excellent ligands due to offering many metal complexes especially group 11–12 metals. The synthesis of gold(I) and silver(I) complexes with chiral phosphorus-1,1,-dithiolate and triphenylphosphine have been described and investigated.

**Conclusions:**

In the present work, we report the synthesis, charactreization of the chiral phosphorus-1,1-dithiolate ligands and preparing the gold(I) and silver(I) phosphorus-1,1-dithiolate or S-donor with phosphine complexes. The molecular structure of the Compound **4** was determined by X-ray diffraction. Due to an easy synthesis method of phosphorus-1,1-dithiolate compounds and a good complexion reagent, it is possible the improvement of the collecting metallic gold or silver from the minerals. When the more ionic salt of phosphorus-1,1-dithiolate compounds were prepared in this way, the water can be used as a cheap solvent. As a result, it can be an alternatively method for the collecting metallic gold or silver from the minerals in future.

## Background

Phosphorus-1,1’-dithiolate ligands are an important class in organophophorus chemistry [1,2]. To date, many phosphorus-1,1’-dithiolate have been synthesized and widely utilised in agricultural, medicinal and technological fields[1-4]. Since the discovery of Lawesson reagent’s and its anologue, they have been used a thionation reagent in organic chemistry and also considerable number of dithiophosphonates and their metal complexes have been synthesized [5-29]. For example, dithiophosphonates which are derivatives of phosphorus-1,1’-dithiolate ligands are not commercially available but they can be easily synthesized by the reaction of Lawesson’s reagent or Ferrocenyl Lawesson’s reagent and the respective alcohols or amines due to a ring opening reaction by nucleophilic attack. The application of metal complexes in the field of medicinal, bioinorganic and bioorganic chemistry has become important in recent decades. For example, some complexes of gold(I), such as Auranofin and some related complexes have been used in the treatment of severe rheumathoidal arthritis [30]. Although there are many gold(I), silver(I) and copper(I) complexes of dithiophosphonates [8-12,22,24], chiral dithiophosphonates and their metal complexes are rare. Recently, a few gold(I) complexes with chiral dithiophosphonates have been reported in our laboratory [5,6].

In the present work, we report mono and dinuclear metal complexes with chiral dithiophosphonates and also triphenylphosphine complexes. All compounds were characterized by elemental analyses, IR, NMR (^1^H-, ^13^C-, ^31^P-) spectroscopy as well as MS measurements.

## Results and discussion

**Scheme 1:**
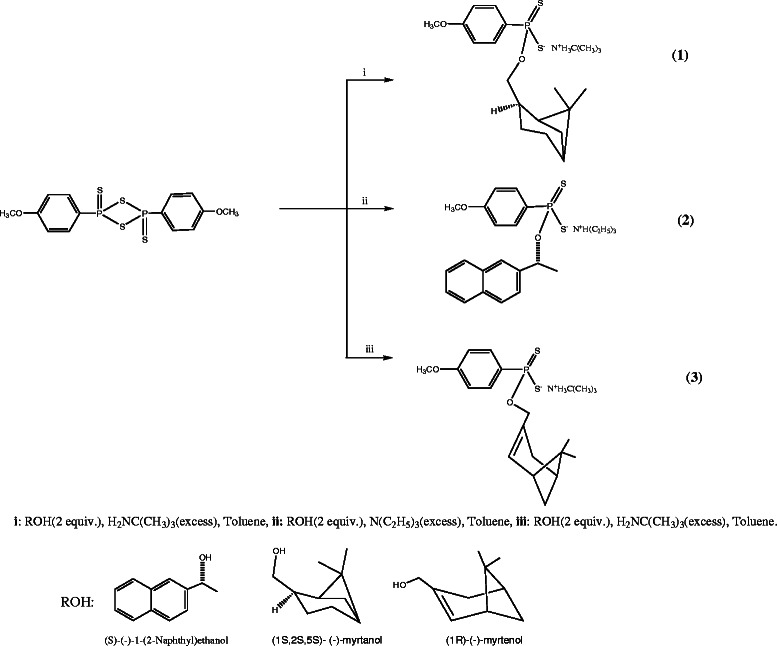
Synthesis of 1-3.

Chiral phosphorus-1,1-dithiolate ligands have been synthesized from Lawesson’s reagent and chiral hydroxyl compounds (Scheme 1).

O-dithiophosphonic acid derivatives of phosphorus-1,1,-dithiolate were formed as liquids and were treated with suitable amine in order to convert them to their salts **1**–**4**. The ^31^P NMR spectr**a** of the ligands **1**–**4** were measured in [D_6_]DMSO and showed one signal as expected. The synthesis and X-ray determination of the compound **2** was reported by Solak et. al. [7] but the spectral studies of the compound **2** were not. All ligands **1**–**4** and gold(I) complexes reported here have been characterised by elemental analysis, IR, NMR and mass spectrocopies. An analogue (triethyl ammonium -O,O^’^-diborneildithiophosphate) of the compound **4** was sythesized by Ohta *et. al.*[31]. In this study, a single crytal structural and full spectroscopically studies of the compound **4**, *tert*-butylammonium -O,O^’^-diborneildithiophosphate, also performed here.

**Scheme 2:**
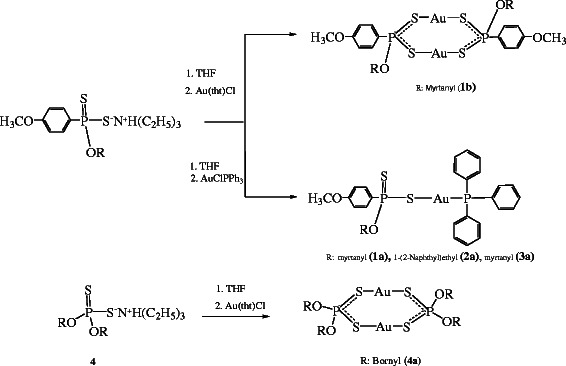
Synthesis of dinuclear and mononuclear gold(I) complexes 1a-4a.

The synthesis of gold(I) and silver(I) complexes with chiral phosphorus-1,1,-dithiolate and triphenylphosphine have been described and also characterized by elemental analyses, IR, NMR and MS spectroscopies (Additional file 1). The synthesis mononuclear and dinuclear gold(I) complexes were summarized in the Scheme 2. The ligands **1**, **2** and **3** were reacted with AuCl(PPh_3_) in order to obtain gold(I)-phosphin complexes with phosphorus-1,1,-dithiolate (Scheme 2). The ^31^P NMR spectrum of **1a** exhibits two signal at 100.33 ppm (PS_2_) and 37.30 ppm (PPh_3_). The complexes **2a** and **3a** also showed two signals in the ^31^P NMR spectrum as expected and other spectroscopic data confirm their structure (Additional file 1). The reaction of the ligands **1** and **4** with Au(tht)Cl gave rise to dinuclear gold(I) complex **1b** and **4a**. The spectra of the complexes **1b** and **4a** were suitable and agree with reported similar structures by Van Zyl et. al. [8-10,22]. To obtain dinuclear gold(I) complexes with amido derivatives of phosphoro-1,1-dithilato, all attempts were unsuccessful. The complex **1b** displayed two signals as expexted in its ^31^P NMR spectrum. One signal is assigned to the trans isomer and the other is assigned to cis isomer. Both signals in the ^31^P NMR spectrum for dinuclear and mononuclear gold(I) complexes are slightly upfield when compared to the free ligands.

**Scheme 3:**
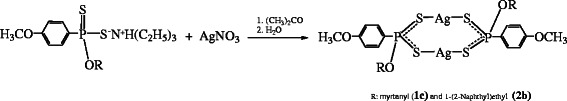
Synthesis of dinuclear silver(I) complexes (1c and 2c).

In the case of dinuclear silver(I) complexes, the ligands were reacted with AgNO_3_ in acetone-water(1:1) at room temperature (Scheme 3). The ^31^P NMR spectra of the dinuclear silver(I) complexes are similar to the dinuclear gold(I) complexes. The expected cis and trans isomer were observed at 107.35 and 105.49 ppm ( for **1c**) and 107.2 and 105.8 ppm (for 2**b**) (Additional file 1).

The IR spectrum of the ligands and their complexes showed two characteristic bands at around 692–642 cm^-1^ and 582 – 515 cm ^-1^ which are assigned to ν_as_(PS_2_) and ν_s_(PS_2_), respectively (Additional file 1). The molecular ion peak of the ligands **2** and 4, the complexes **1b** and 2**a** were observed at m/z = 430 (for [M + 1]^+^), 476 [for M]^+^, 832 (for [M]^+^), 1104 (for [M]^+^), respectively, in their mass spectra whereas the other ligands and the complexes exhibited m/z-values for identifiable certain fragments (Additional file 1). Specific rotations of all compounds showed that only one optical isomer was formed.

### Molecular structure of 4

**Figure 1:**
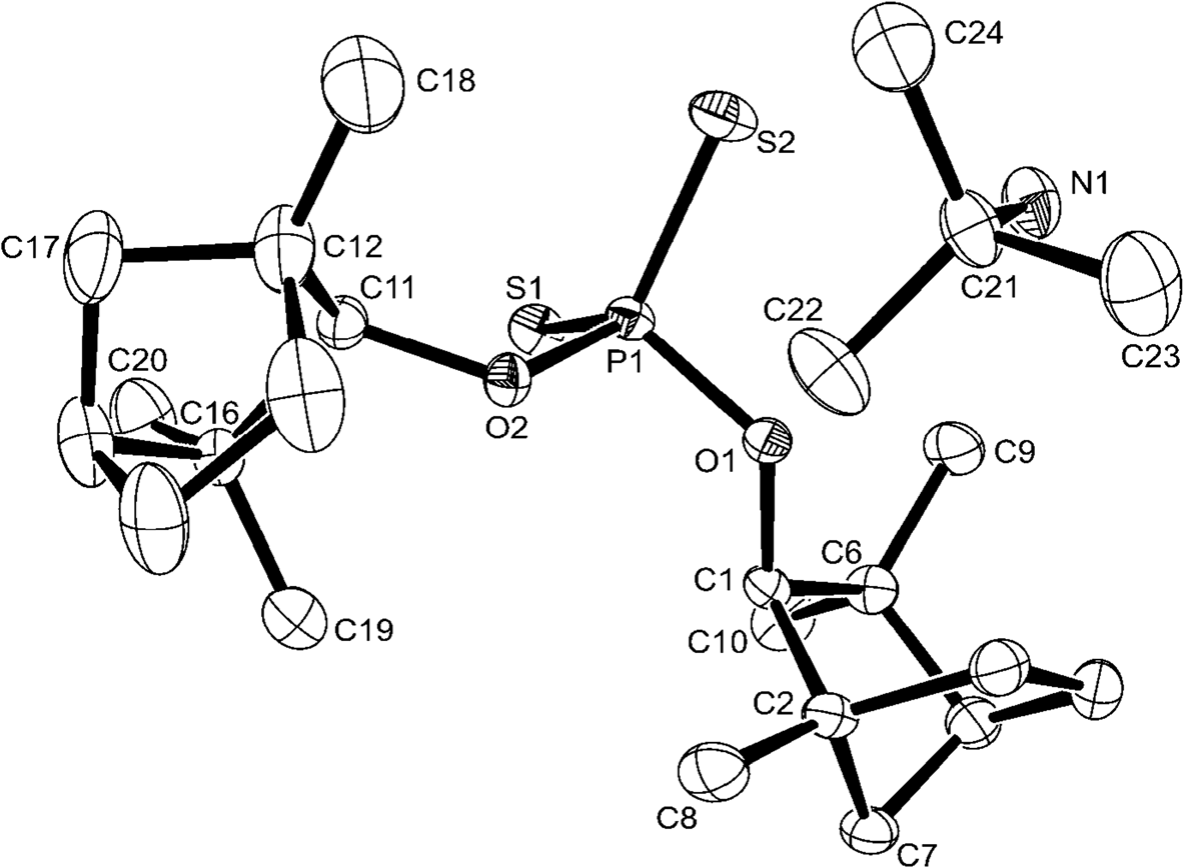
Molecular structure of 4 (ORTEP, 50% probability).

Single crystals of **4** suitable for X-ray diffraction studies were obtained from acetone/*n*-hexane. Compound **4** crystallizes in the orthorhombic space group P2(1)2(1)2(1) with Z = 4. The molecular structure of **4** is depicted in Figure 1. Selected bond lengths and angles are given Table 1 and Crystal and structure-refinement data in Table 2. The P1-S1 and P1-S2 bond lengths are 197.56(5) and 198.13(5) pm, respectively. Those values are very close to each other due to a delocalized PS_2_ fragments. Bond lengths and angles are in a good agreement with those of related previously reported compounds [7,9]. Figure 1 shows that the phosphorus atom has a tetrahedral coordination environment with [O1-P1-S1 113.30(4)°, O2-P1-S1 110.12(4)°, O(1)-P(1)-S(2) 105.02(4)°, O2-P1-S2 110.05(4)°] and the O1-P1-O2 [99.61(6)°] having a deviation from the ideal value (109.5°).

**Table 1:** Bond lengths [pm] and angles [°] for 4

	
S(1)-P(1)	197.56(5)
S(2)-P(1)	198.13(5)
P(1)-O(1)	159.14(11)
P(1)-O(2)	160.96(11)
O(1)-P(1)-O(2)	99.61(6)
O(1)-P(1)-S(1)	113.30(4)
O(2)-P(1)-S(1)	110.12(4)
O(1)-P(1)-S(2)	105.02(4)
O(2)-P(1)-S(2)	110.05(4)
S(1)-P(1)-S(2)	117.23(2)

**Table 2:** Crystal data and structure refinement for 4

		
Empirical formula	C24 H46 N O2 P S2	
Formula weight	475.71	
Temperature	130(2) K	
Wavelength	71.073 pm	
Crystal system	Orthorhombic	
Space group	P2(1)2(1)2(1)	
Unit cell dimensions	a = 1029.440(10) pm	α= 90°
	b = 1366.64(2) pm	β= 90°
	c = 1949.83(2) pm	δ = 90°
Volume	2.74316(6) nm3	
Z	4	
Density (calculated)	1.152 Mg/m3	
Absorption coefficient	0.272 mm-1	
F(000)	1040	
Crystal size	0.5 × 0.4 × 0.3 mm3	
Theta range for data collection	2.98 to 30.51°	
Index ranges	−14 < =h < =14, -19 < =k < =19, -27 < =l < =27	
Reflections collected	56268	
Independent reflections	8360 [R(int) = 0.0375]	
Completeness to theta = 30.51°	99.8%	
Absorption correction	Semi-empirical from equivalents	
Max. and min. transmission	1 and 0.98374	
Refinement method	Full-matrix least-squares on F2	
Data / restraints / parameters	8360 / 0 / 390	
Goodness-of-fit on F2	0.997	
Final R indices [I > 2sigma(I)]	R1 = 0.0348, wR2 = 0.0909	
R indices (all data)	R1 = 0.0403, wR2 = 0.0925	
Absolute structure parameter	0.01(5)	
Largest diff. peak and hole	0.626 and −0.309 e.Å-3	

### Experimental

## Materials

When necessary, the reactions were carried out under an atmosphere of nitrogen using standard Schlenk techniques. All other chemicals were purchased from commercial sources and used directly without further purification.

### Measurements

Elemental analyses were determined with a GmbH varioMICRO CHNS apparatus. Melting points were determined by using Electrotermal apparatus. NMR spectra were recorded on a Bruker AVANCE DRX 400 NMR spectrometer and Jeol GSX 270 in CDCl_3_ and d_6_-DMSO. IR spectra was measured on a Perkin-Elmer 2000 FTIR spectrophotometer (4000 – 400 cm^-1^). Mass spectra were recorded with an AGILENT 1100 MSD and Waters machines. Optical rotation values were determined with an automatic digital ADP 440+ polarimeter.

### X- ray crystallograpy

Data were collected on an Xcalibur-S diffractometer (Agilent Technologies) using Mo-K_α_ radiation (λ = 71.073 pm) and ω-scan rotation (see Table 2). Data reduction was performed with CrysAlis Pro [32] including the program SCALE3 ABSPACK for empirical absorption correction. The structure was solved by direct methods and the refinement of all non-hydrogen atoms was performed with SHELX97 [33]. All non-hydrogen atoms were refined with anisotropic thermal parameters. With the exception of one borneol molecule (C(11) to C(20)) all hydrogen atoms were located on difference Fourier maps calculated at the final stage of the structure refinement. The structure figure was generated with DIAMOND-3 [34]. Crystallographic details are given in the Additional file 2. CCDC (**4**) 921954 contains the supplementary crystallographic data for this paper. The data can be obtained free of charge from The Cambridge Crystallographic Data Centre via http://www.ccdc.cam.ac.uk/data_request/cif.

### Synthesis of compounds

#### Synthesis of ^*t*-^butyl ammonium salt of (1S,2S,5S)-(−)-myrtanyl −4- methoxyphenyl dithiophoshonate 1

2,4-Bis(4-methoxyphenyl)-1,3,2,4-dithiadiphosphetane-2,4-disulfide (Lawesson’s reagent: LR) ( 0.50 g, 1.23 mmol) was reacted with 1S,2S,5S)-(−)-myrtanol (0.39g, 2.4693 mmol) in toluene (20 mL). The mixture was refluxed until all solids had dissolved. The colourless solution was cooled to rt, filtered and treated with excess tert-butyl amine. The product precipitated at −18°C from toluene as a white solid, which was isolated by filtration, washed with toluene and n-hexane and then dried in air. Yield: 0.76 g (72%), m.p.: 161-163°C. [α]_589_^25^ = 57.14 (c = 0.105 in THF). Elemental analysis calculated for C_21_H_36_NO_2_PS_2_ (429.63 g.mol^-1^):C, 58.71; H, 8.45; N, 3.26; S, 14.93, Found: C, 59.18; H, 8.51; N, 3.18; S, 14.69. IR(cm^-1^): 670 (ν_asym_ PS_2_) and 556(ν_sym_ PS_2_). ^1^H NMR (CDCl_3_): δ = 8.07 (dd, 2H, ^3^J_P,H_ = 13.65 Hz, ^3^J_H,H_ = 8.80 Hz), 6.9 (dd, 2H, ^4^J_P.H_ =2.57 Hz, ^3^J_H.H_ =8.75 Hz), 3.85 (s, 3H, OCH3), 3.48 ( m. 2H, OCH_2_), 1.88 (m, 7H, myrtanyl), 1.37 (s, 9H.3×CH3), 1.17 (d, 3H, CH_3_), 0.79(d, 3H, CH_3_) ppm. ^13^C NMR (CDCl_3_): δ =161.32 (d, ^4^J_P,C_ =2.91 Hz), 133.16(d, ^1^J_P,C_ =109.49 Hz), 132.32 (d, ^2^J_P,C_ =13.43 Hz), 112.91(d, ^3^J_P,C_ =14.83 Hz), 68.78(d, ^2^J_P,C_ =8.55), 55.36, 54.00, 35.45(d, ^3^J_P,C_ = 8.40 Hz) 28.22, 42.10, 40.86, 39.02, 26.63, 24.09, 23.36, 20.12, 18.12 ppm. ^31^P NMR (CDCl_3_): δ = 105.24 ppm. MS: m/z = 430[M + 1]^+^. 357[M-NH_2_C(CH_3_)_3_]^+^.

### Triethyl ammonium salt of (S)-(−)-O-(2-naphthyl)ethyl-4-methoxyphenyl dithiophosphonate 2

Compound **2** was prepared as described in the literature [7]. [α]_589_^25^ = −37.74 (c = 0.053 in THF). Elemental analysis calculated for C_25_H_34_NO_2_PS_2_ (475.66 g.mol^-1^): C, 63.13; H, 7.21;. N, 2.95; S, 13.48 %, Found: C, 63.59; H, 7.32; N, 2.92; S, 12.74 %. IR(cm^-1^): 667 (ν_asym_ PS_2_) and 569 (ν_sym_ PS_2_). ^1^H-NMR (CDCl_3_): δ = 9.89 (br, H, HN), 8.1 (dd, 2H, ^3^J_P,H_ = 13.58 Hz, ^3^J_H,H_ = 8.81 Hz), 7.21 (m. 4H.), 7.53 (dd, 1H), 7.40 (m. 2H), 6.7 (dd, 2H. ^4^J_P,H_ =2.62 Hz, ^3^J_H,H_ =8.87 Hz), 5.7 (m. 1H, OCH, ^3^J_P,H_ = 13.07, ^2^J_H,H_ = 6.53 Hz), 3.69 (s, 3H, OCH3), 3.07 (q, 6H, 3xNCH_2_), 1.56 (d, 3H, CH_3_), 1.18 (t, 9H, 3×CH_3_) ppm. ^13^C NMR (CDCl_3_): δ = 160.75(d, ^4^J_P,C_ =3.03 Hz), 141.64(d, ^3^J_P,C_ = 4.32 Hz), 135.54(d, ^1^J_P,C_ =111.36 Hz), 133.11, 132.10, 132.03(d, ^2^J_P,C_ =13.48 Hz), 127.97, 127.50, 125.70, 125.45, 124.99(d, ^4^J_P,C_ =5.01 Hz_._), 112.52(d, ^3^J_P,C_ =14.88 Hz), 73.84(d, ^2^J_P,C_ =7.41), 55.23, 45.90, 24.66(d, ^3^J_P,C_ =4 .19 Hz), 8.48 ppm. ^31^P NMR (CDCl_3_): δ = 105.35 ppm.

### Synthesis of ^*t*-^Butyl ammonium salt of (1R)-(−)-O-myrtenyl-4- methoxyphenyl dithiophoshonate 3

Compound **3** was prepared in the same way as compound **1**, from LR (0.50 g, 1.23 mmol) and (1R)-(−)-myrtenol (0.38 mL. 2.4693 mmol ) in toluene (20 mL). Yield: 0.73 g (69%), m.p.: 110-113°C. [α]_589_^25^ = 159.1 (c = 0.044 in THF). Elemental analysis calculated for C_21_H_34_NO_2_PS_2_ (427.61 g.mol^-1^): C, 58.99; H, 8.01; N, 3.28; S, 15.00; Found: C, 59.18; H, 8.51; N, 3.18; S, 14.69. IR(cm^-1^): 669 (ν_asym_ PS_2_) and 556 (ν_sym_ PS_2_). ^1^H NMR (CDCl_3_): δ = 8.07 (dd, ^3^J_P,H_ = 13.59 Hz, ^3^J_H,H_ = 8.83 Hz), 6.9 (dd, 2H, ^4^J_P,H_ =2.66 Hz, ^3^J_H,H_ =8.88 Hz), 4.1 (m, 2H, OCH_2_), 5.46 (t, H, CH), 3.84 (s, 3H, OCH_3_), 2.2 (m, 5H, myrtenyl ), 1.38 (s, 9H, 3×CH_3_), 1.27 (d, 3H, CH_3_) 1.09 (d, 1H, CH) 0.74 (d, 3H, CH_3_) ppm. ^13^C NMR (CDCl_3_): δ =161.33(d, ^4^J_P,C_ =3.03 Hz), 144.20(d, ^3^J_P,C_ =9.68 Hz), 133.25 (d, ^1^J_P,C_ =109.67 Hz), 132.29 (d, ^2^J_P,C_ =13.50 Hz), 119.69, 112.92 (d, ^3^J_P,C_ =14.91 Hz), 67.70 (d, ^2^J_P,C_ = 7.62), 55.36, 53.17, 43.25, 40.76, 37.98, 31.35, 28.19, 26.16, 20.97 ppm. ^31^P NMR (CDCl3): δ =104.88 ppm.

### Synthesis of ^t-^Butyl ammonium salt of O,O^’^-dibornyl-4-methoxyphenyl dithiophosphonate (4)

P_4_S_10_ (1g, 2.25 mmol) was reacted with (−) borneol (2.77 g, 9 mmol) in a 1:4 ratio in 50 mL hot toluene to give the crude dithiophosphoric acid. The reactions were refluxed until all solids dissolved and the yellow solutions were obtained. The solution was filtered and then was treated with excess *tert*-butyl amine at r.t. The *tert*-butyl ammonium salt of O,O^’^-dibornyl-dithiophosphoric acids resulted in as a precipitated white solid product. The product was filtrated, washed with pentane several times, dried under vacuum and recrystallized from acetone/hexane. Yield: 3.89g (91%), m.p.: 188°C. Elemental analysis calculated for C_24_H_46_NOPS_2_(475.73 g/mol): C, 60.79; H, 9.26; N, 2.89; S, 13.18; Found: C, 60.59; H, 9.74; N, 2.94; S 13.48. IR(cm^-1^): 665 (ν_asym_ PS_2_) and 560 (ν_sym_ PS_2_). ^1^H-NMR (CDCl_3_): δ = 4.64 (t, 2H), 2.28 (br, 4H), 2.00 (br, 2H), 1.71-179(br, m, 4H), 1.23(br, m, 4H), 0.91(s, 6H, 2×CH_3_), 0.86(s, 6H, 2×CH_3_), 0.85(s, 6H, 2×CH_3_). ^13^C-NMR (CDCl_3_): δ = 53.83, 49.52(d, ^2^J_P,C_ = 6.74 Hz), 47.31, 44.93, 37.44, 28.30, 27.00, 19.94, 18.69, 13.67. ^31^P-NMR (CDCl_3_): δ = 106.53(d). MS (FAB): m/z 476 [M]^+^.

### Synthesis of complexes

#### **[Au(PPh**_**3**_**)(R**^**1**^**PS**_**2**_**(OR**^**2**^**)] (R**^**1**^**: 4-methoxyphenyl and R**^**2**^**: ((1S,2S,5S)-(−)-myrtanyl) 1a**

A solution of the compound 1 (0.044 g. 0.102 mmol ) in THF (10 mL) was added dropwise to a solution of AuClPPh_3_ (0.05 g, 0.102 mmol) in THF (10 mL) and stirred at r.t. for 2 h. A colourless solution was observed and then a solid, t-butylammonium chloride, was immediately precipitated. The reaction mixture was filtered and the solvent was removed under reduced pressure. The reaction mixture was filtered and 10 mL of n-hexane was added to the solution. The solvent was removed at room temperature and a white crystalline product was isolated. The white crystalline product was dried in air. Yield: 0.042 g (51%), m.p.: 72–74°C. [α]_589_^25^ = 37.97 (c = 0.079 in THF). Elemental analysis calculated for C_35_H_39_O_2_SP_2_Au (814.78 g.mol^-1^); calcd: C, 51.60; H, 4.82; S, 7.87; Found: C, 49.78; H, 4.52; S, 10.61. IR(cm^-1^): 668(ν_asym_ PS_2_) and 536(ν_sym_ PS_2_). ^1^H NMR (CDCl_3_): δ = 7.89(dd, 2H, ^3^J_P,H_ = 13.77 Hz, ^3^J_H,H_ = 8.61 Hz), 7.50(m, 15H,), 6.77 (dd, 2H, ^3^J_H.H_ = 8.31 Hz, ^3^J_P,H_ = 2.18 Hz), 3.95 (q, H, OCH_2_), 3.73 (s, 3H, OCH_3_) 1.80 - 0.78 (br, 15H, myrtanyl) ppm. ^31^P NMR (CDCl_3_): δ = 100.33 (PS_2_), 37.31(PPh_3_) ppm.

#### **[Au{R**^**1**^**PS**_**2**_**(OR**^**2**^**)}]**_**2**_** (R**^**1**^**: 4-methoxyphenyl and R**^**2**^**: (1S,2S,5S)-(−)-myrtanyl) 1b**

A solution of **1** (0.15 g, 0.35 mmol) in THF (10 mL) was added dropwise to a solution of Au(tht)Cl (tht = tetrahydrothiophene) ( 0.11 g, 0.35 mmol) in THF (10 mL) and stirred for 2 h. A solid, t-butylammonium chloride, was immediately observed. The reaction mixture was filtered and 10 mL of n-hexane was added to the mixture. The solvent was removed at room temperature and orange crystals were isolated. Yield: 0.097 g (50%), m.p.: 123–125°C. [α]_589_^25^ = 29.85 (c = 0.067 in THF). Elemental analysis calculated for C_34_H_48_O_4_S_4_P_2_Au_2_ (1104.90 g.mol^-1^): C, 36.96; H, 4.38; S, 11.61; Found: C, 36.76; H, 4.51; S, 11.75. IR(cm^-1^): 640(ν_asym_ PS_2_) and 542(ν_sym_ PS_2_). ^1^H NMR (CDCl_3_): δ = 7.91 (br, 4H ), 6.90 (dd, 4H, ^3^J_H,H_ = 8.63 Hz, ^3^J_P,H_ = 2.41 Hz), 4.40 (br, 4H, OCH_2_), 3.79 (s, 6H, OCH_3_), 1.81-1.29 (m, 18H. myrtenyl), 1.17 (s, 6H, 2×CH_3_), 0.79 (s, 6H, 2xCH_3_) ppm. ^13^C NMR (CDCl_3_): δ = 163.06(d, ^4^J_P,C_ = 3.12 Hz), 133.87 (d, ^1^J_P,C_ =118.49 Hz), 132.46 (d, ^2^J_P,C_ = 13.25 Hz), 113.95 (d, ^3^J_P,C_ =15.43 Hz), 71.31(d, ^2^J_P,C_ =5.33), 55.51, 35.68 (d, ^3^J_P,C_ = 7.70 Hz), 42.23, 40.84, 39.36, 28.20, 26.71, 24.03, 23.75, 20.28, 18.09 ppm. ^31^P NMR (CDCl_3_): (cis, trans isomer) δ = 104.12, 100.95. **MS**(ESI): m/z = 1104[M]^+^, 907[M-Au]^+^.

#### **[Ag{R**^**1**^**PS**_**2**_**(OR**^**2**^**)}]**_**2 **_**(R**^**1**^**: 4-methoxyphenyl and R**^**2**^**: (1S,2S,5S)-(−)-myrtanyl)** 1**c**

The compound **1** (0.20 g, 0.41 mmol) was dissolved in acetone (10 mL). A solution of AgNO_3_ (0.08 g, 0.41 mmol) in a mixture (10 mL) of acetone-water (1:1) was added dropwise to the solution and stirred for 2 h. A white powder solid was obtained, filtered and recrytallized in CHCl_3_. The solvent was removed at room temperature and white powder were isolated.Yield: 0.14g (73%), m.p.: 124–126°C. [α]_589_^25^ = 27.39 (c = 0.073 in CHCl_3_). Elemental analysis calculated for C_34_H_48_O_4_S_4_P_2_Ag_2_ (926.70 g.mol^-1^): C, 44.07; H, 5.22; S, 13.84; Found: C, 43.62; H, 5.32; S, 13.37. IR(cm^-1^): 656 (ν_asym_ PS_2_) and 543 (ν_sym_ PS_2_). ^1^H NMR (CDCl_3_): δ = 7.90 (dd, 4H, ^3^J_P,H_ = 13.15 Hz, ^3^J_H,H_ = 8.54 Hz), 6.84 (br, 4H), 3.76 (s, 6H, 2×OCH_3_), 1.9 (m, 18H, myrtanyl), 1.11 (d, 6H, CH_3_) 0.73 (d, 6H, CH_3_) ppm. ^13^C NMR (CDCl_3_): δ = 162.24, 132.50 (d, ^2^J_P,C_ = 13.24 Hz), 129.83 (d, ^1^J_P.C_ = 119.83 Hz), 113.54 (d, ^3^J_P,C_ = 15.06 Hz), 70.25 (d, ^2^J_P,C_ = 7.03), 55.42, 35.57 (d, ^3^J_P,C_ = 8.40 Hz**)**, 42.19, 40.87, 39.22, 26.69, 24.11, 23.66, 20.54, 18.14 ppm. ^31^P NMR (CDCl_3_) (cis, trans isomer) δ = 107.35 and 105.49 ppm.

#### **[Au(PPh**_**3**_**)(R**^**1**^**PS**_**2**_**(OR**^**2**^**)] (R**^**1**^**: 4-methoxyphenyl, R**^**2**^**: ((S)-(−)-O-(2-naphthyl)ethyl) 2a**

**2a** was prepared in a similar manner to **1a** using AuClPPh_3_ (0.049 g. 0.10 mmol) and t-butyl ammonium ((S)-(−)-O-(2-naphthyl)ethyl −4-methoxyphenydithiophosphonate **2** (0.049 g. 0.10 mmol ). Yield: 0.04g (48%), m.p.: 62–64 °C. [α]_589_^25^ = 51.94 (c = 0.077 in THF). Elemental analysis calculated for C_37_H_33_O_2_S_2_P_2_Au (832.72 g.mol^-1^): C, 53.37; H, 3.99; S, 7.70; Found: C, 52.50; H, 4.12; S, 7.12 %. IR(cm^-1^): 674 (ν_asym_ PS_2_) and 537 (ν_sym_ PS_2_). ^1^H NMR (CDCl_3_): δ = 8.09(dd, 2H, ^3^J_P,H_ = 13.94 Hz, ^3^J_H,H_ = 8.86 Hz), 7.5(br, 15H), 6.79 (dd, 2H, ^3^J_H.H_ = 8.86, ^3^J_P,H_ = 3.06 Hz), 6.10(q, H, OCH), 3.78 (s, 3H, OCH_3_), 1.78 (m, 3H, CH_3_) ppm. ^13^C NMR (CDCl_3_): δ = 161.52 (d, ^4^J_P,C_ =4.42 Hz), 135.21(d, ^1^J_P.C_ =134.60 Hz), 134.23(d, ^2^J_P,C_ =13.94 Hz), 132.07, 131.93, 131.70, 129.15, 128.14, 127.88, 127.56, 127.45, 125.84, 125.66, 125.01, 124.77, 124.66, 113.14 (d, ^3^J_P,C_ =15.63 Hz), 74.66(d, ^2^J_P.C_ = 6.61 Hz), 55.30, 24.82(d) ppm. ^31^P NMR (CDCl_3_): δ = 100.43 (PS_2_), 37.20 (PPh_3_). MS(ESI) = m/z: 832[M]^+^.

#### **[Ag{R**^**1**^**PS**_**2**_**(OR**^**2**^**)}]**_**2**_** (R**^**1**^**: 4-methoxyphenyl and R**^**2**^**: (2-naphthyl)ethyl) 2b**

**2b** was prepared in a similar manner to **1b** using AgNO_3_ (0.07 g, 0.41 mmol) and triethyl ammonium(S)-(−)-O-(2-naphthyl)ethyl-4-methoxyphenyl dithiophosphonate, (0.20 g, 0.41 mmol). Yield: 0.13 g (64%), m.p.: 114–115°C. [α]_589_^25^ = −36.14 (c = 0.083 in CHCl_3_). Elemental analysis calculated for C_38_H_36_0_4_P_2_S_4_Ag_2_ (962.65 g.mol^-1^): C, 47.41; H, 3.77; S, 13.32; Found: C, 46.15; H, 3.99; S, 12.29. IR(cm^-1^): 655 (ν_asym_ PS_2_) and 569 (ν_sym_ PS_2_). ^1^H NMR (CDCl_3_): δ = 7.60 (br, 18H), 6.55 (br, 4H), 5.62 (br, 2H, OCH), 3.55 (s, 6H, OCH_3_), 1.50 (d, 6H, CH_3_) ppm. ^13^C NMR (CDCl_3_): δ = 162.06 (d, ^4^J_P,C_ = 2.26 Hz), 139.78 (d, ^4^J_P,C_ = 4.27 Hz), 133.01 (d, ^2^J_P,C_ = 11.68 Hz), 129.94 (d, ^1^J_P,C_ =117.92 Hz), 128.26, 128.16, 127.03, 125.89, 124.69. 124.99 (d, ^4^J_P,C_ = 5.01 Hz_._), 113.22 (d, ^3^J_P,C_ = 12.57 Hz), 55.27, 24.66 (d) ppm. ^31^P NMR (CDCl_3_) (cis, trans isomer): δ = 107.21, 105.84 ppm. MS (ESI) = m/z: 855[M-Ag]^+^.

#### **2.3.6. [Au(PPh**_**3**_**)(R**^**1**^**PS**_**2**_**(OR**^**2**^**)] (R**^**1**^**: 4-methoxyphenyl and R**^**2**^**: (1R-(−)-myrtenyl) 3a**

**3a** was prepared in a similar manner to **1a** using AuClPPh_3_ (0.05 g. 0.10 mmol) and t-butyl ammonium-(1R)-(−)-myrtenyl-4-methoxyphenyldittiophosphonate **3** (0.048 g, 0.10 mmol). Yield: 0.036 g. (43%), m.p.: 76–78°C. [α]_589_^25^ = 30.76 (c = 0.065 in THF). C_35_H_37_O_2_SP_2_Au (812.73 g.mol^-1^); calcd: C, 51.72; H, 4.59; S, 7.89%, found: C, 51.01; H, 5.22; S, 6.69%. IR(cm^-1^): 691 (ν_asym_ PS_2_) and 565 (ν_sym_ PS_2_). ^1^H NMR (CDCl_3_): δ = 7.99 (dd, 2H, ^3^J_P.H_ = 13.84 Hz, ^3^J_H.H_ = 8.76 Hz), 7.41-7.19(br, 15H, ), 6.75 (dd, 2H, ^3^J_H,H_ = 8.79 Hz, ^3^J_P,H_ = 2.96 Hz), 5.49 (t, H, CH), 4.52 (q, H, OCH_2_), 3.71 (s, 3H, OCH_3_), 2.25 - 078 (br, 12H, myrtenyl) ppm. ^13^C NMR (CDCl_3_): δ = 161.56 (s, ^4^J_P,C_ = 3.32 Hz), 144.22 (d, ^3^J_P,C_ = 9.87 Hz), 134.37(d, ^1^J_P.C_ = 121.39 Hz), 132.29 (d, ^2^J_P,C_ =13.50 Hz), 131.75, 129.52, 128.94, 120.26, 113.25 (d, ^3^J_P.C_ = 15.47 Hz), 66.96 (d, ^2^J_P,C_ = 6.27 Hz), 55.34, 43.32, 40.86, 38.10, 31.20, 26.19, 21.20 ppm. ^31^P NMR (CDCl_3_): δ = 100.60 (PS_2_), 37.29 (PPh_3_) ppm.

#### **Synthesis of [O,O’-(Bornyl)**_**2**_**PS**_**2**_**]Au**_**2**_** 4a**

Compound 4**a** was prepared in a similar manner to **1b** using **4** (0.075 g, 0.155 mmol) and Au(tht)Cl (tht = tetrahydrothiophene) (0.05 g, 0.155 mmol) in THF(20 mL). Yield: 0.065 g (% 70), m.p. 212°C. Elemental analysis calculated for C_40_H_68_O_4_P_2_S_4_Au_2_(1197.10 g/mol): C, 40.67; H, 5.68; S, 10.68; Found C, 40.13; H, 5.72; S, 10.71. IR(cm^-1^): 644(ν_asym_ PS_2_) and 543(ν_sym_ PS_2_). ^1^H-NMR (CDCl_3_): δ = 4.95 (br, t, 2H), 2.43 (br, t, 2H), 1.79(br, s, 4H), 1.44(t, 4H, ^3^J_H,H_ = Hz), 1.30(br, t, 4H), 0.99(s, 6H, 2xCH_3_), 0.93(s, 6H, 2×CH_3_), 0.89(s, 6H, 2xCH_3_). ^13^C-NMR (CDCl_3_): δ = 49.94, 47.55, 47.95, 37.35, 30.05, 27.96, 26.86, 19.91, 18.93, 13.71. ^31^P-NMR (CDCl_3_): δ = 102.19(s). MS (ESI): m/z 999.5 [M-Au]^+^, 967.5[M-AuS]^+^.

## Conclusion

The new chiral phosphorus-1,1-dithiolate ligands were synthesized and were then utilised in the preparation of gold(I) and silver(I) phosphorus-1,1-dithiolate or S-donor with phosphine complexes. If the phosphorus-1,1-dithiolate ligands carefully utilised in the preparation of gold(I) complexes, those type compounds can be improvement of the collecting metallic gold or silver from the minerals. We hope that this study will extend the unexplored area in phosphoro-1,1-dithiolate chemistry.

## Competing interests

The authors declare that they have no competing interests.

## Authors’ contributions

MK has coordinated the experimental work, characterized the structure of the all compounds and wrote the manuscript. SS synthesized the compounds and measured the IR spectra. CA synthesized and obtained the single crystals of the compound **4**. PL carried out X-ray studies. All authors have read and approved the final manuscript.

## Authors’ information

Part of M.Sc. of S. Solak.

## Supplementary Material

Additional file 1 Spectra of the compounds. Click here for file

Additional file 2 Cif file of compound 4. Click here for file
