# Supplementary material for: Novel Gold(I) and Silver(I) Complexes of Phosphorus-1,1,-dithiolates and Molecular Structure of [O,O’-(Bornyl)2PS2]H3NC(CH3)3
Source: Chem Cent J. 2013 May 20;7:89. doi: 10.1186/1752-153X-7-89 (PMC3663823; doi:10.1186/1752-153X-7-89)
Supplement: Additional file 1 — Spectra of the compounds. [file 1752-153X-7-89-S1.pdf]

1. Synthesis of [t]-butyl ammonium salt of (1S,2S,5S)-(-)-myrtanyl -4-methoxyphenyl dithiophoshonate 1

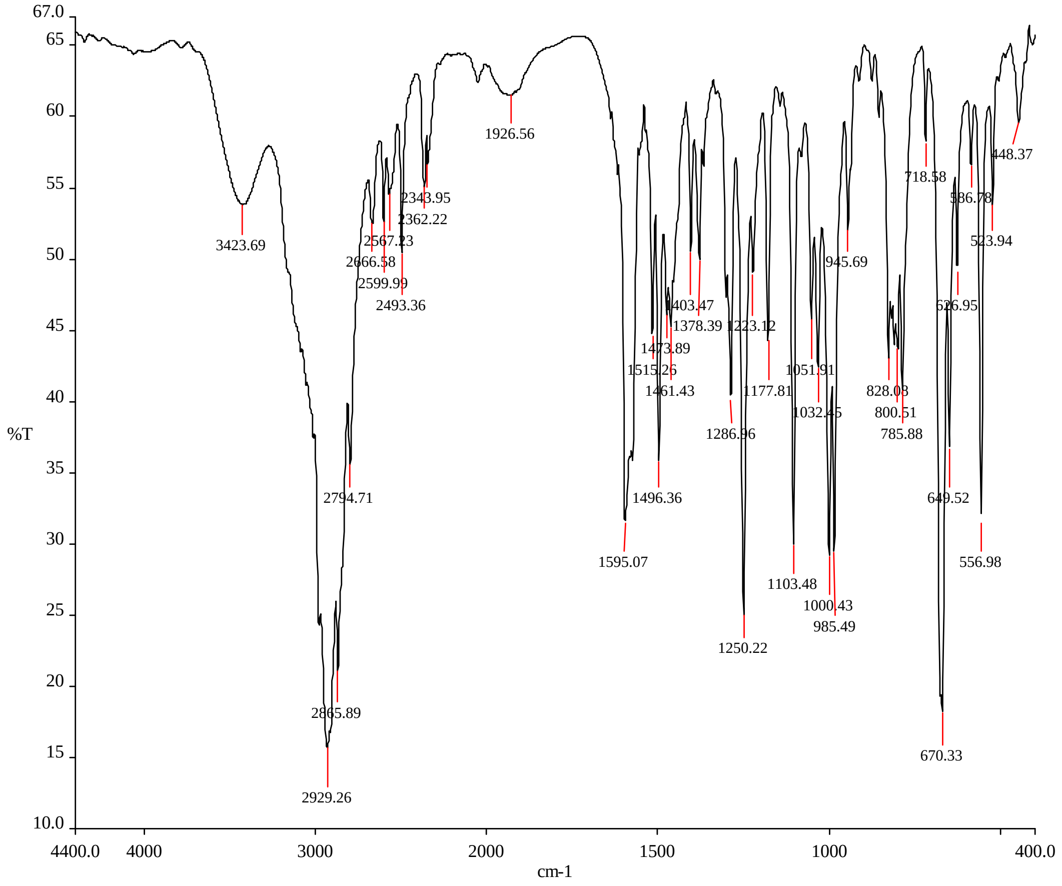

Fig.1 IR spectra of the compound 1

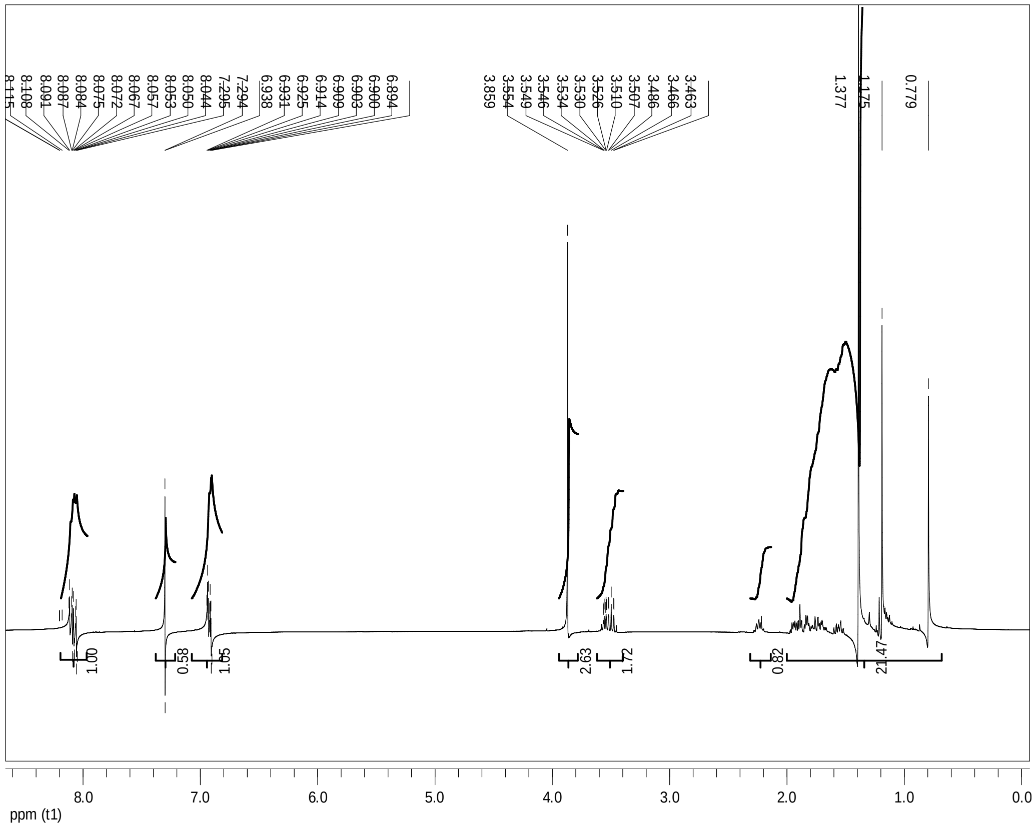

Fig.2. ^{1}H-NMR spectra of the compound 1

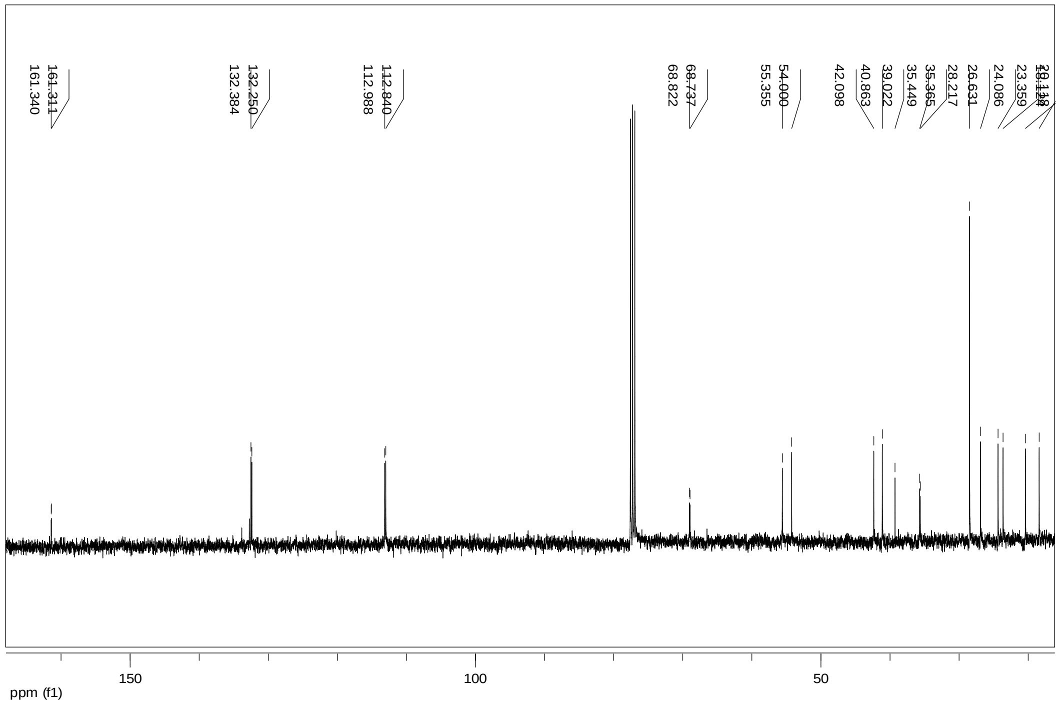

Fig. 3. ^{13}C-NMR spectra of the compound 1

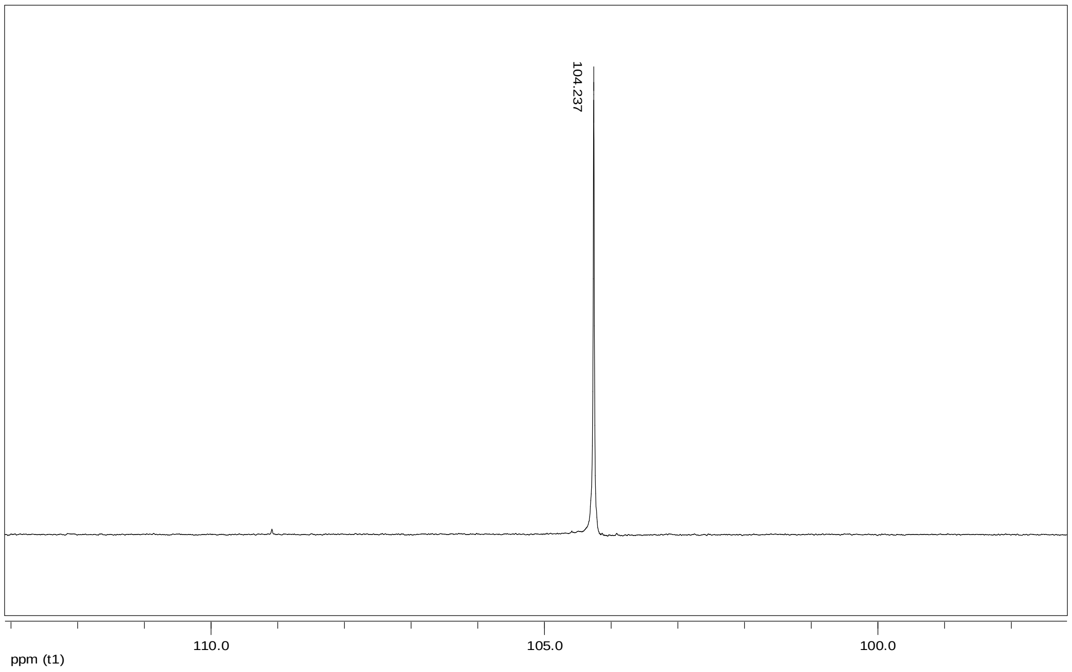

Fig.4. ^{31}P-NMR spectra of the compound 1

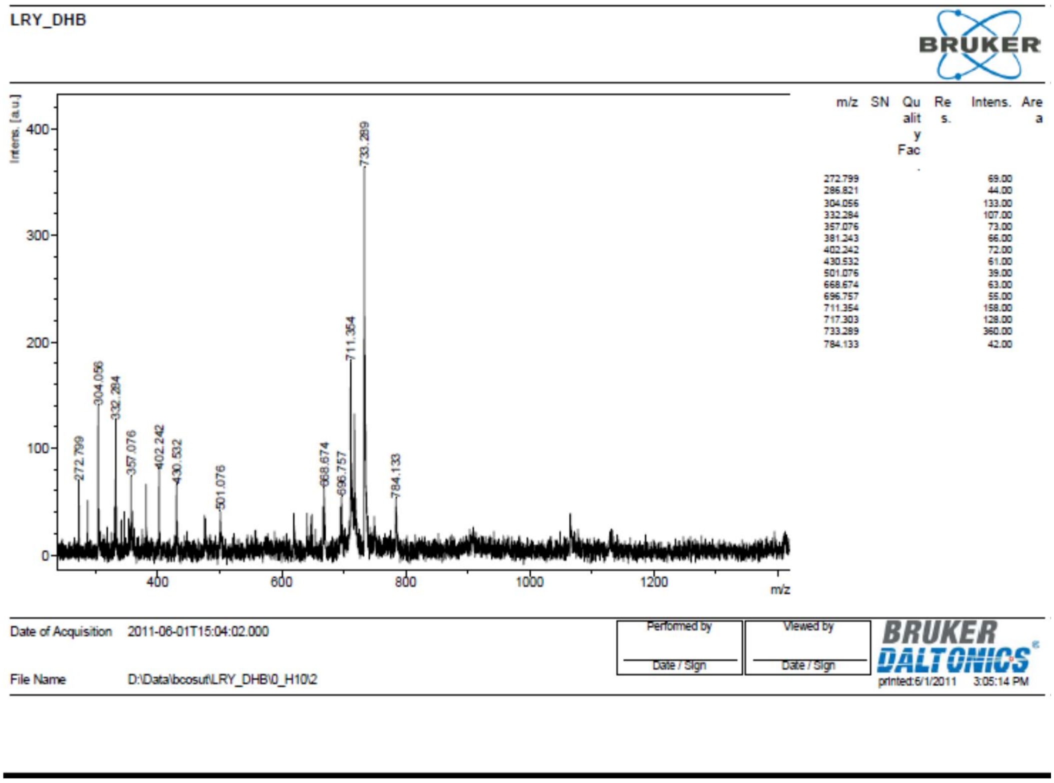

Fig. 5. Mass spectra of the compound 1

2. Triethyl ammonium salt of (S)-(-)-O-(2-naphthyl)ethyl-4-methoxyphenyl dithiophosphonate 2

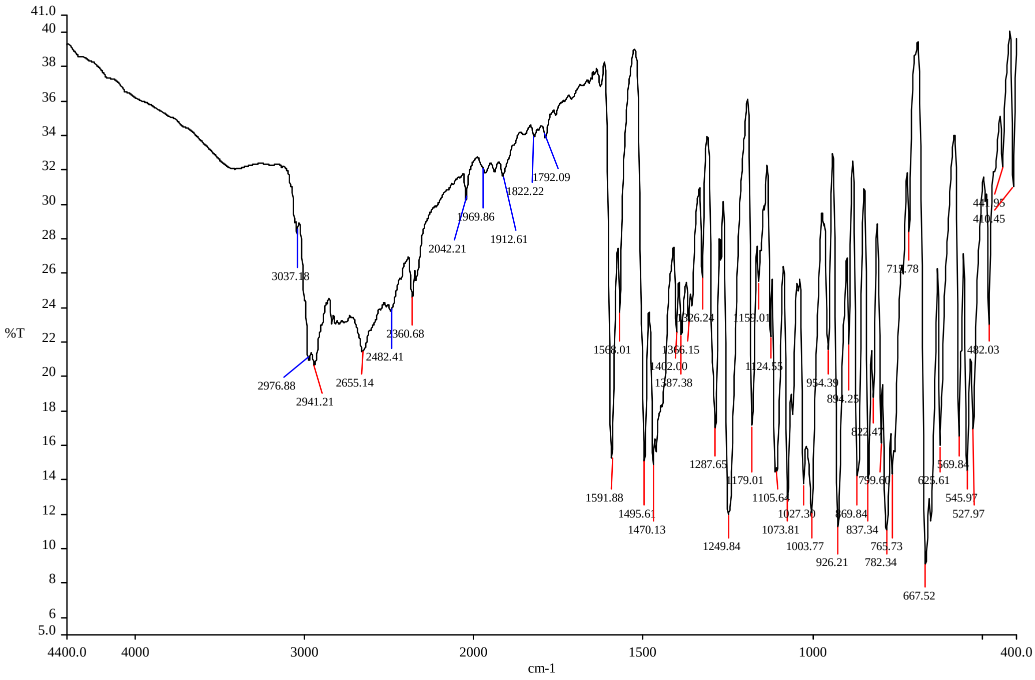

Fig. 6. IR spectra of the compound 2

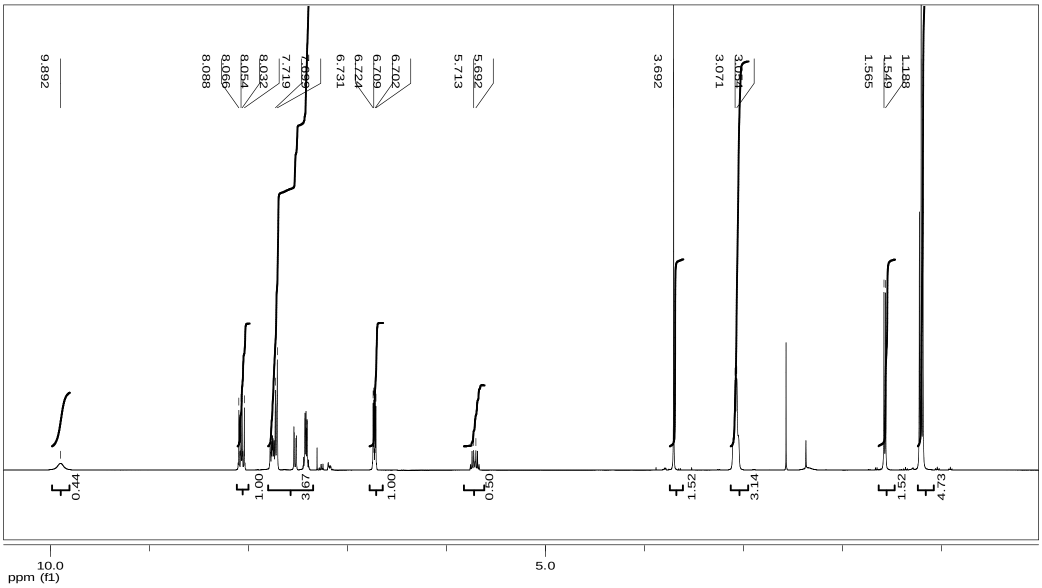

Fig. 7. ^{1}H-NMR spectra of the compound 2

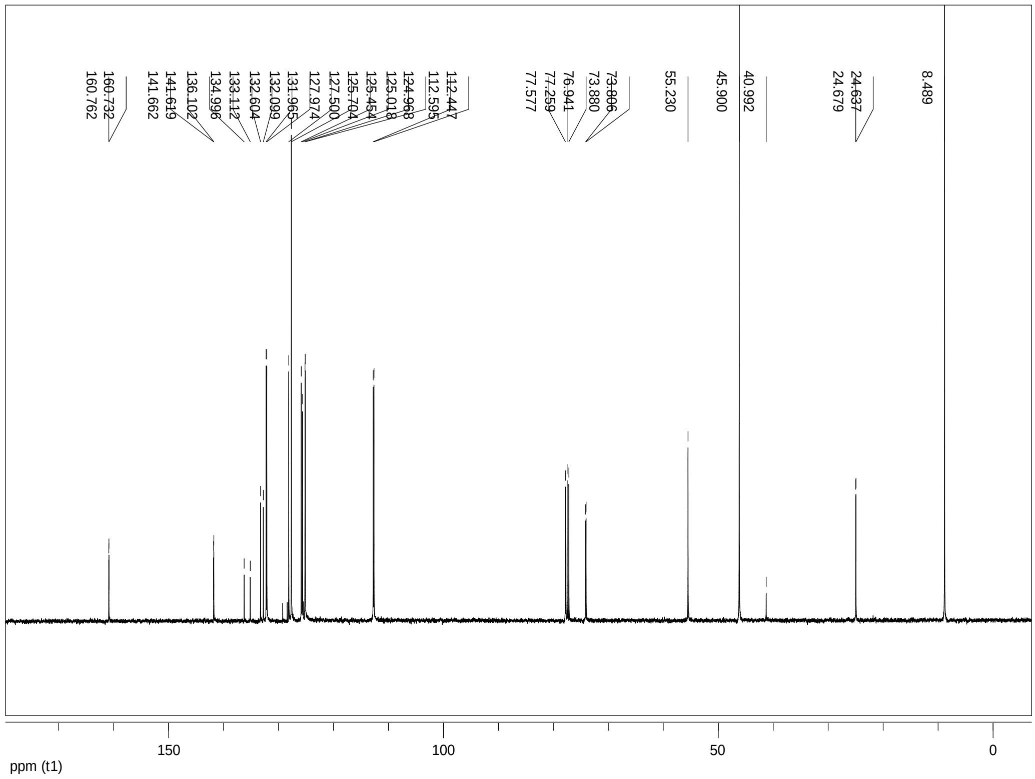

Fig.8. ^{13}C-NMR spectra of the compound 2

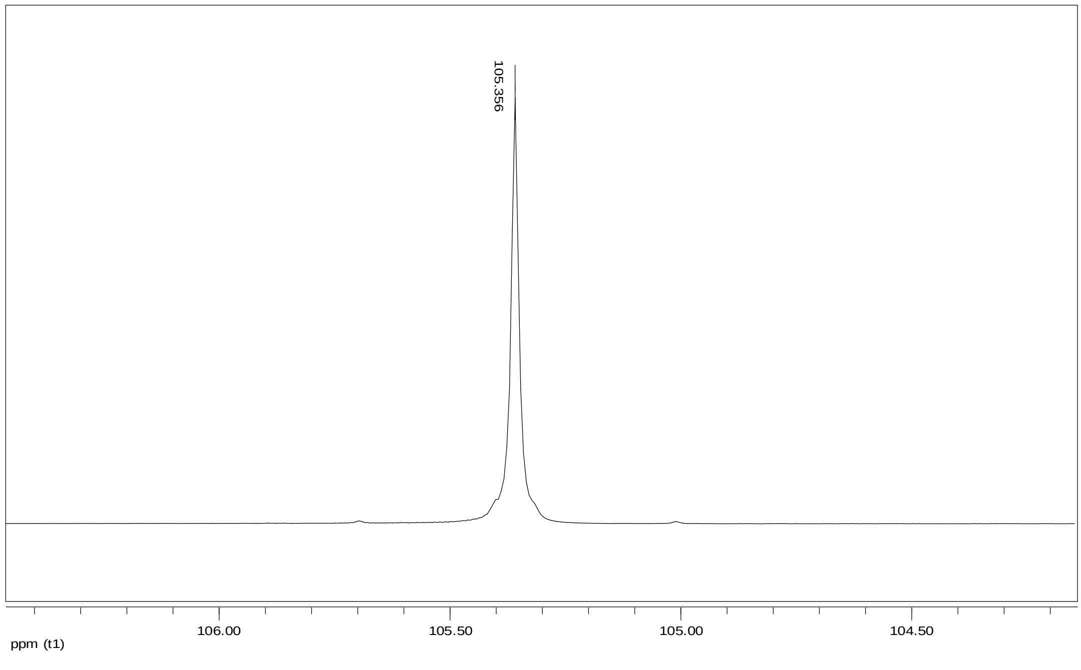

Fig. 9. ^{31}P-NMR spectra of the compound 2

3. Synthesis of ^{t}Butyl ammonium salt of (1R)-(-)-O-myrtenyl-4-methoxyphenyl dithiophoshonate 3

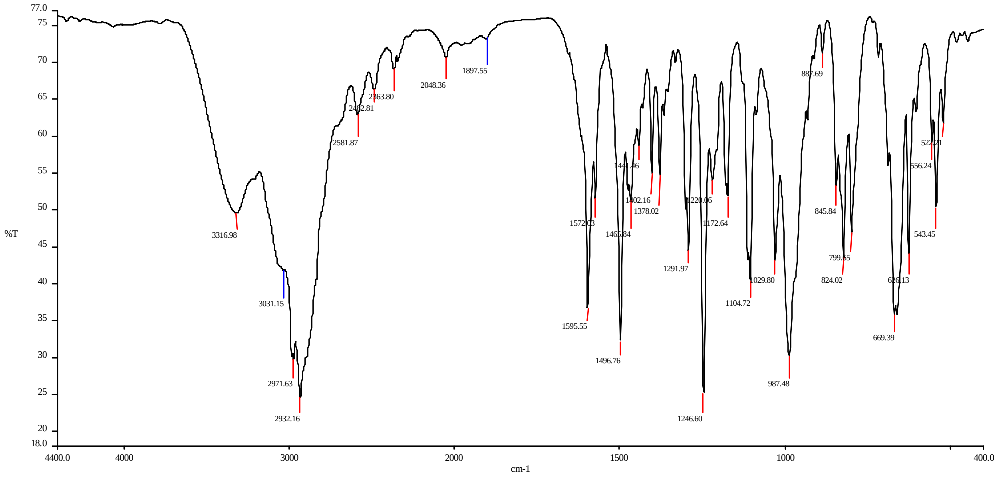

Fig. 10. IR spectra of the compound 3

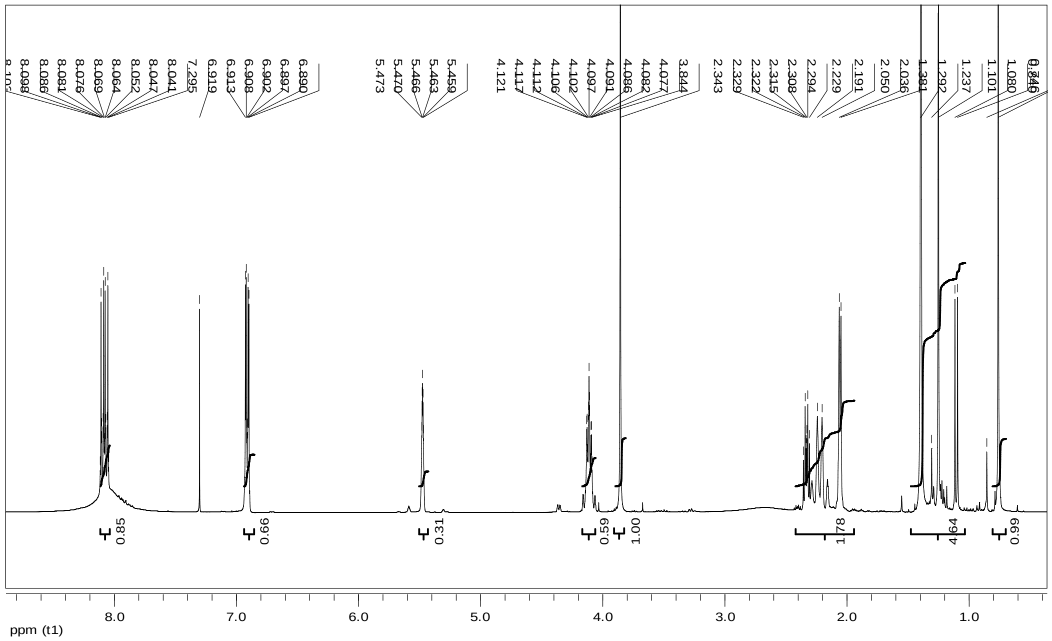

Fig. 11. ^{1}H-NMR spectra of the compound 3

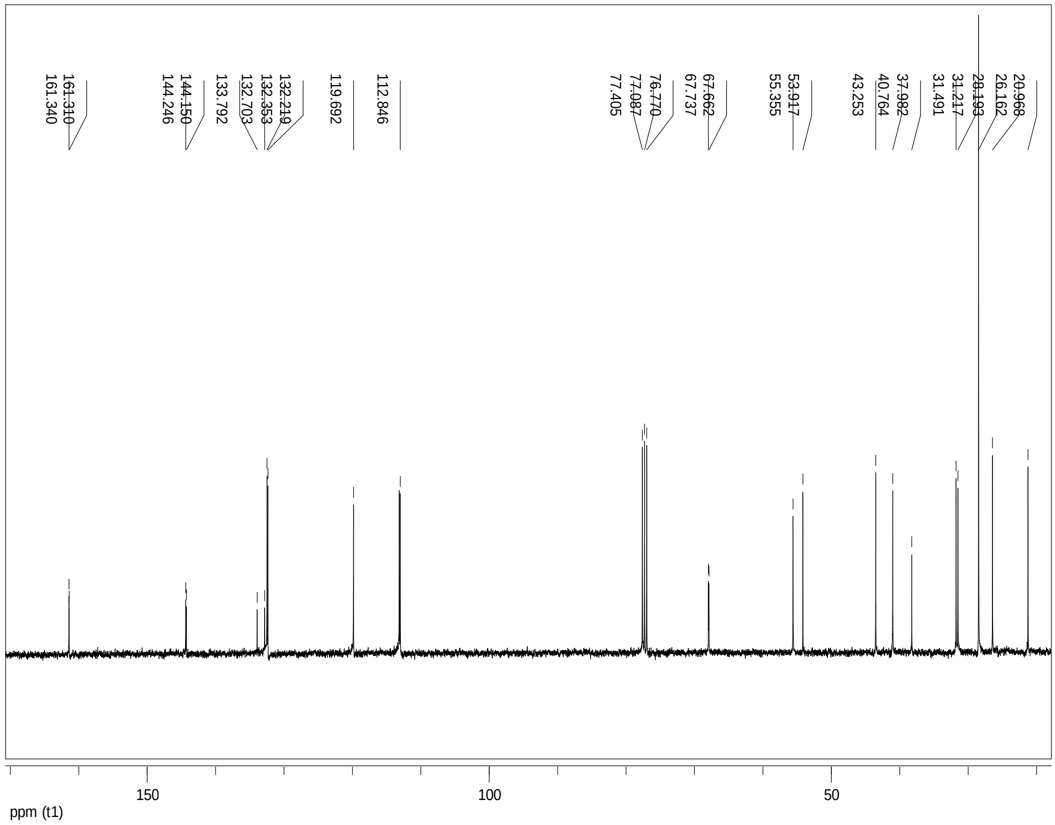

Fig. 12. ^{13}C-NMR spectra of the compound 3

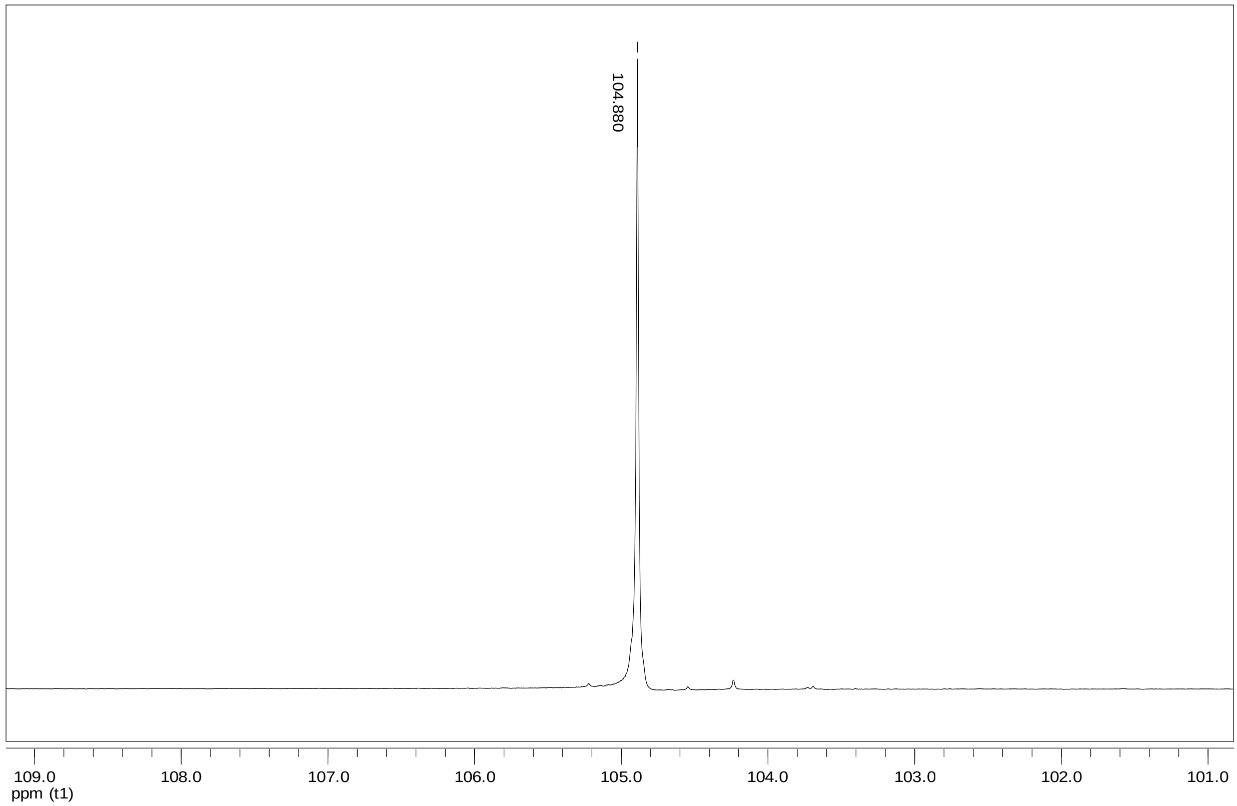

Fig. 13. ^{31}P-NMR spectra of the compound 3

4. Synthesis of $^{t-}$butyl ammonium salt of O,O$^{\hat{0}}$-dibornyl-4-methoxyphenyl dithiophosphonate (4)

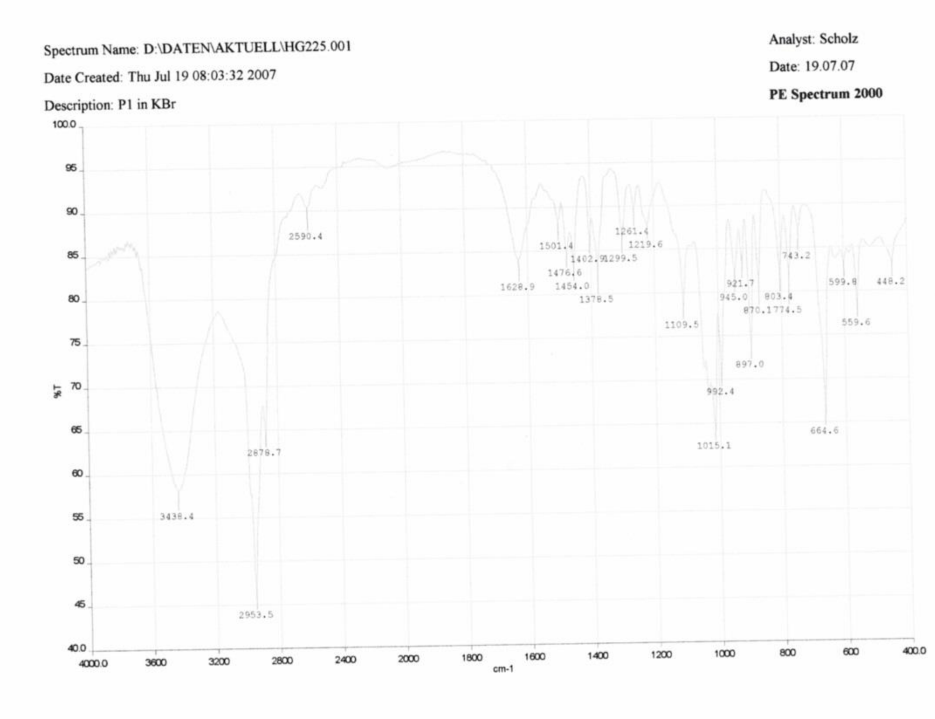

Fig. 14. IR spectra of the compound 4

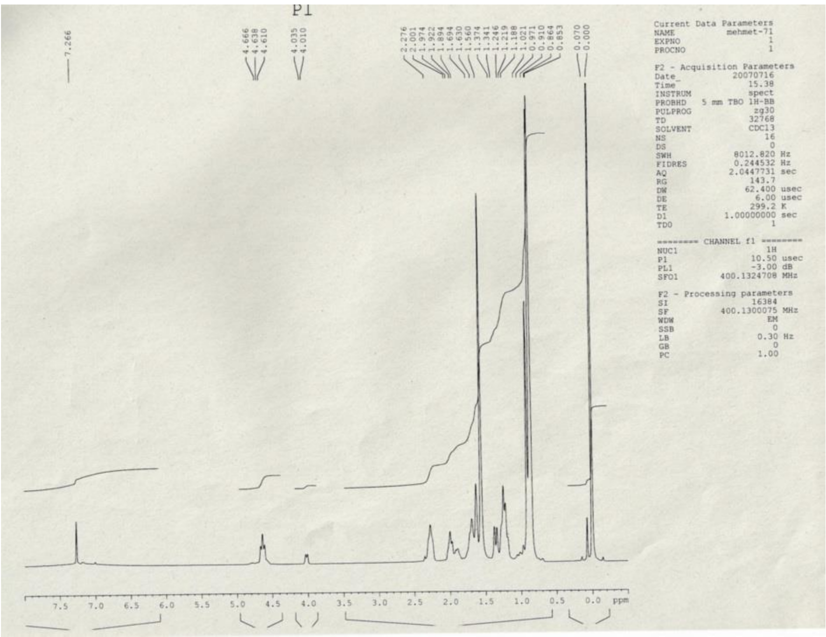

Fig. 15. ^{1}H-NMR spectra of the compound 4

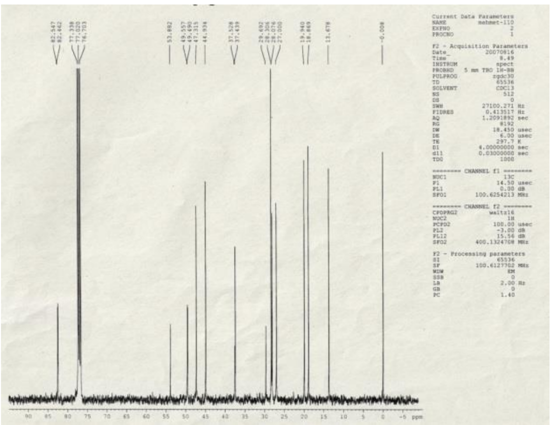

Fig. 16. ^{13}C-NMR spectra of the compound 4

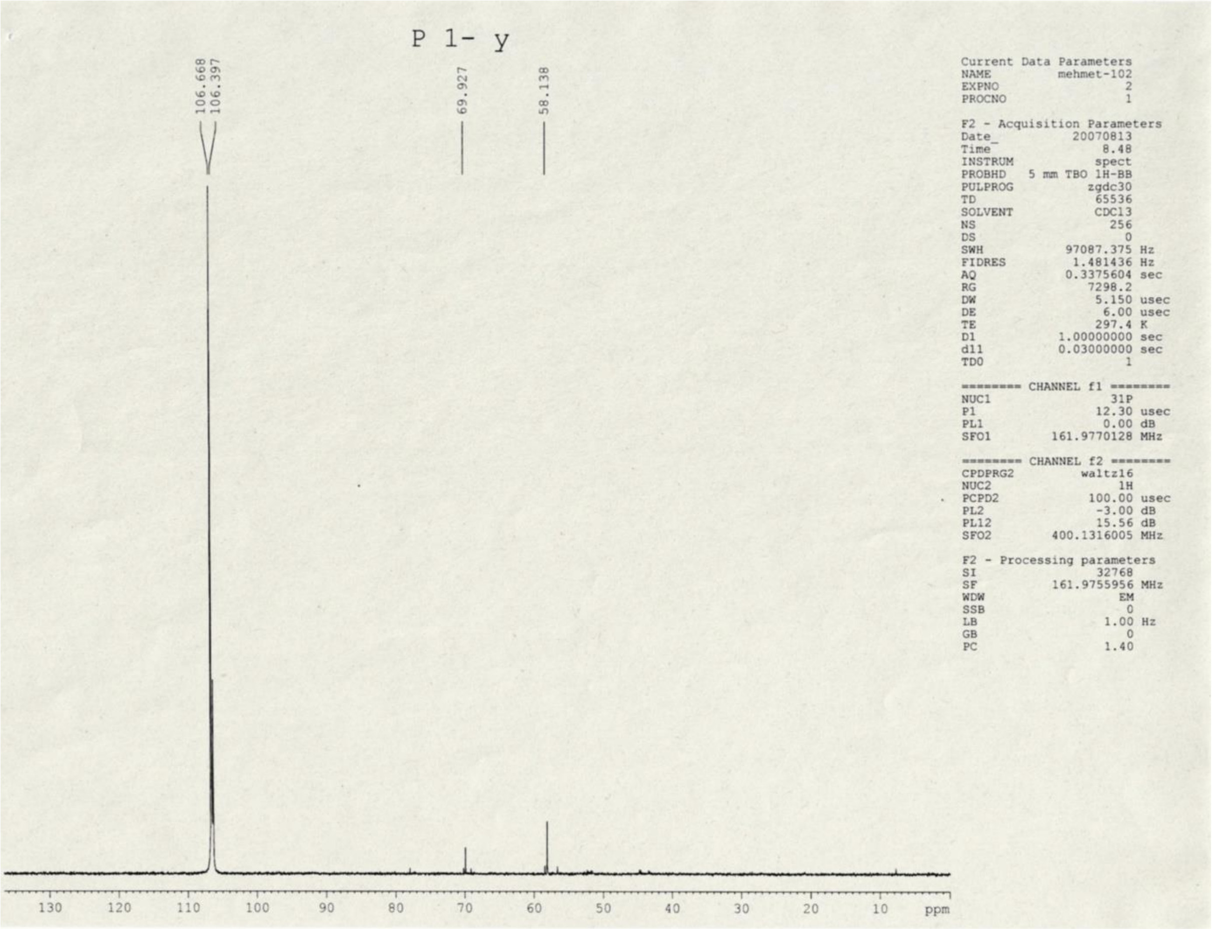

Fig. 17. ^{31}P-NMR spectra of the compound 4

5. [Au(PPh$_3$)(R^{1}PS$_2$(OR^2)] (R^1: 4-methoxyphenyl and R^2: ((1S,2S,5S)-(-)-myrtanyl) 1a

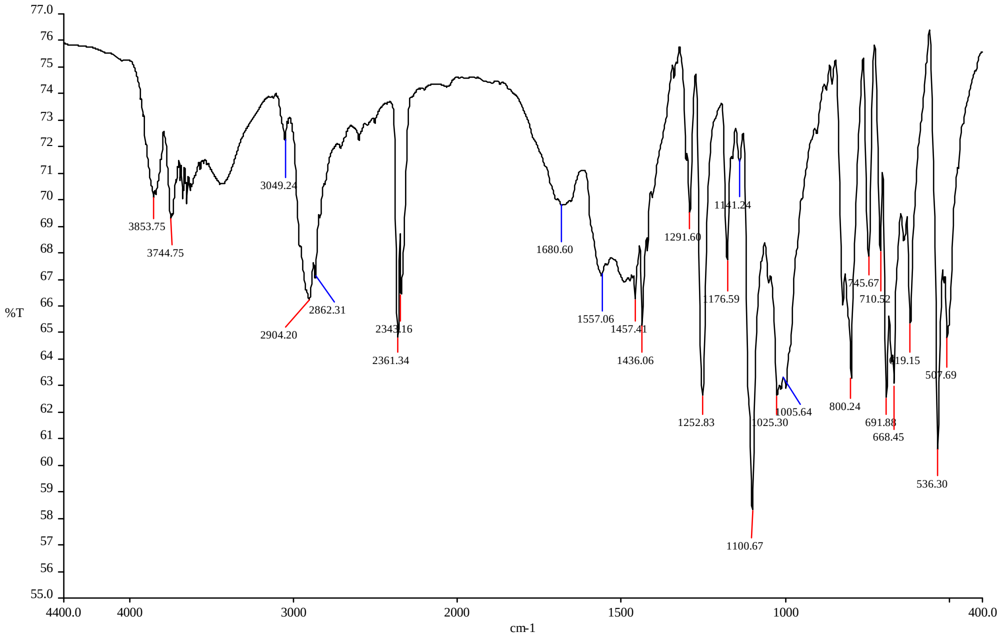

Fig. 18. IR spectra of the compound 1a

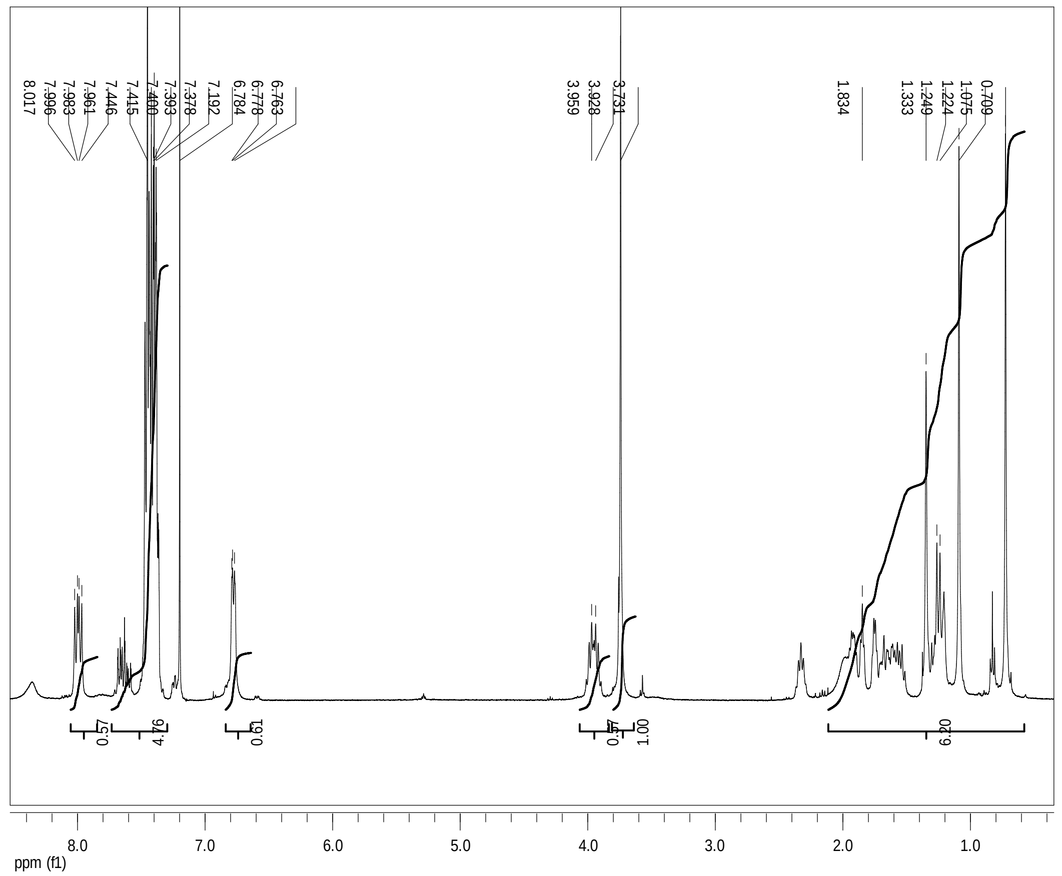

Fig. 19. ^{1}H-NMR spectra of the compound 1a

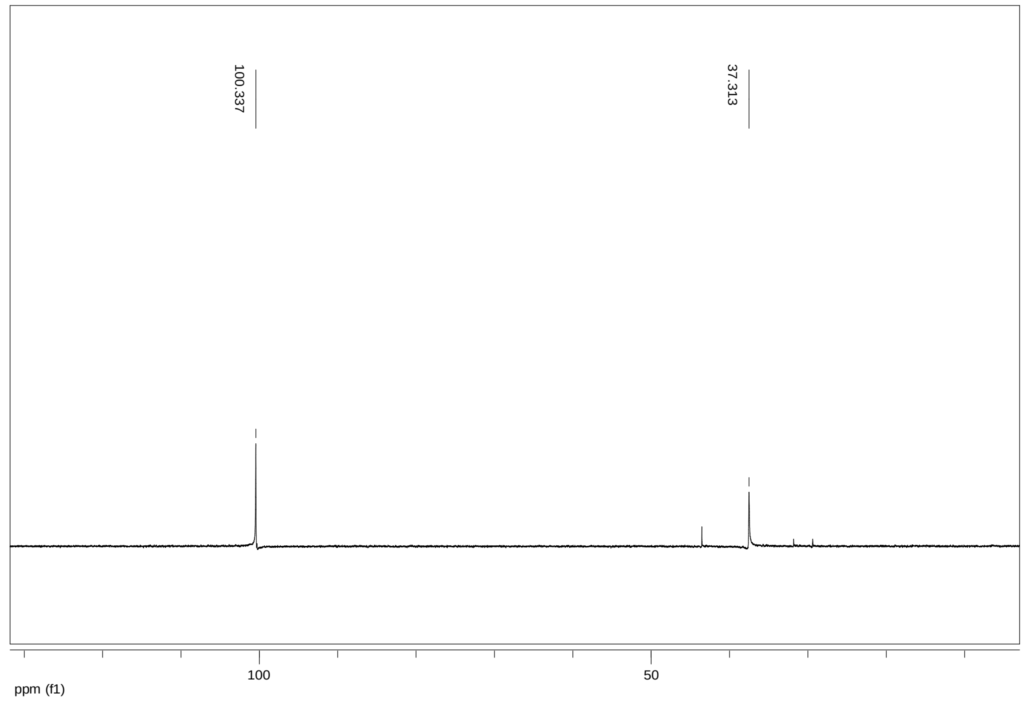

Fig. 20. ^{31}P-NMR spectra of the compound 1a

6. [Au{R[1]PS2(OR[2])}]2 (R[1]: 4-methoxyphenyl and R[2]: (1S,2S,5S)-(-)-myrtanyl) 1b

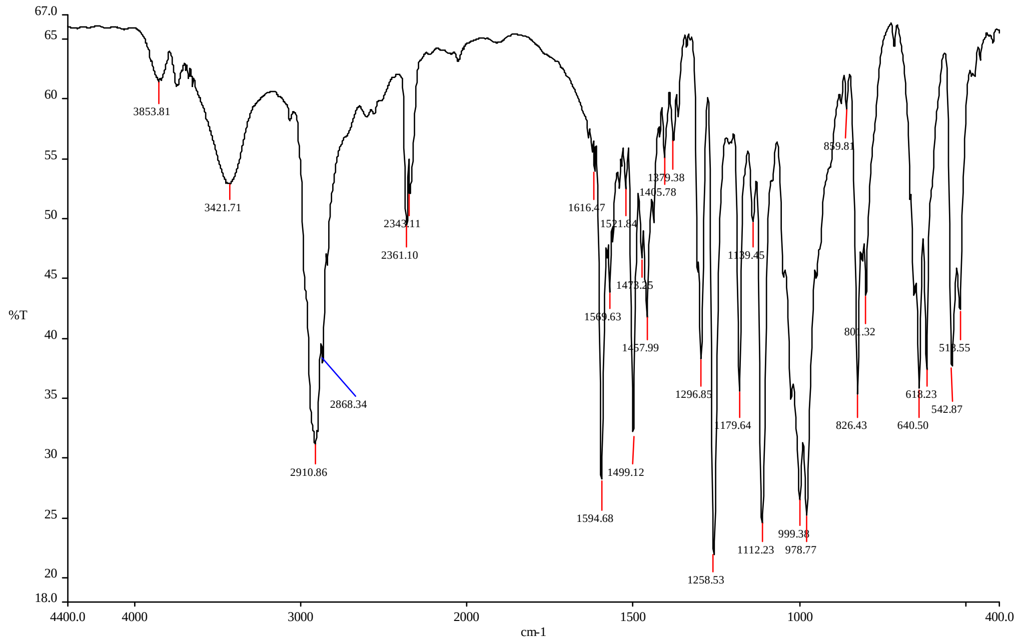

Fig. 21. IR spectra of the compound 1b

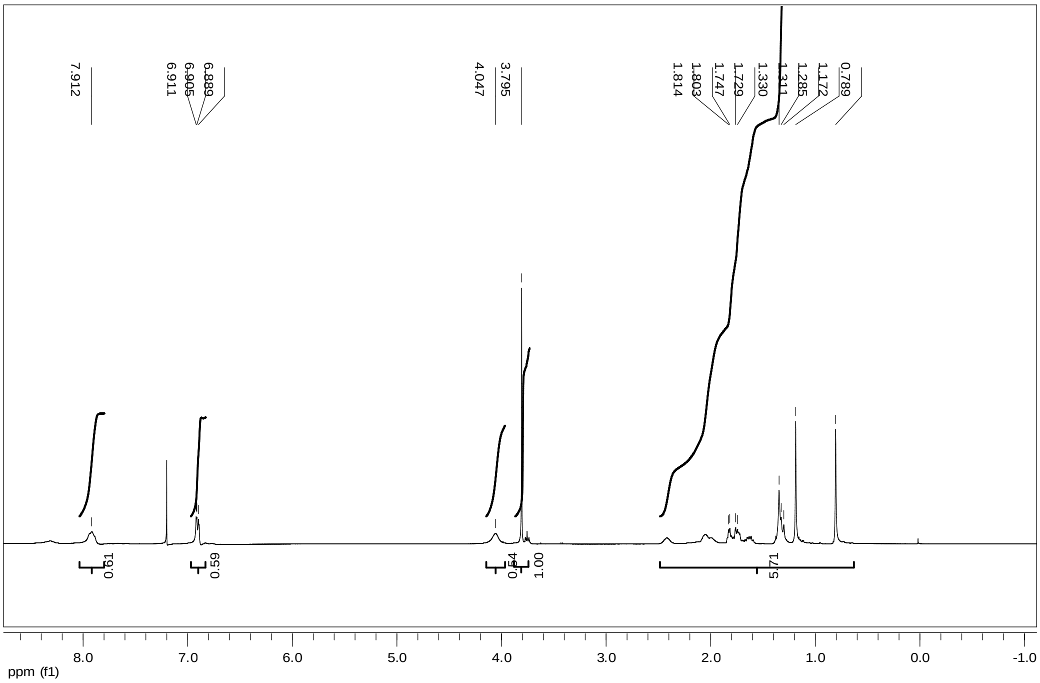

Fig. 22. ^{1}H-NMR spectra of the compound 1b

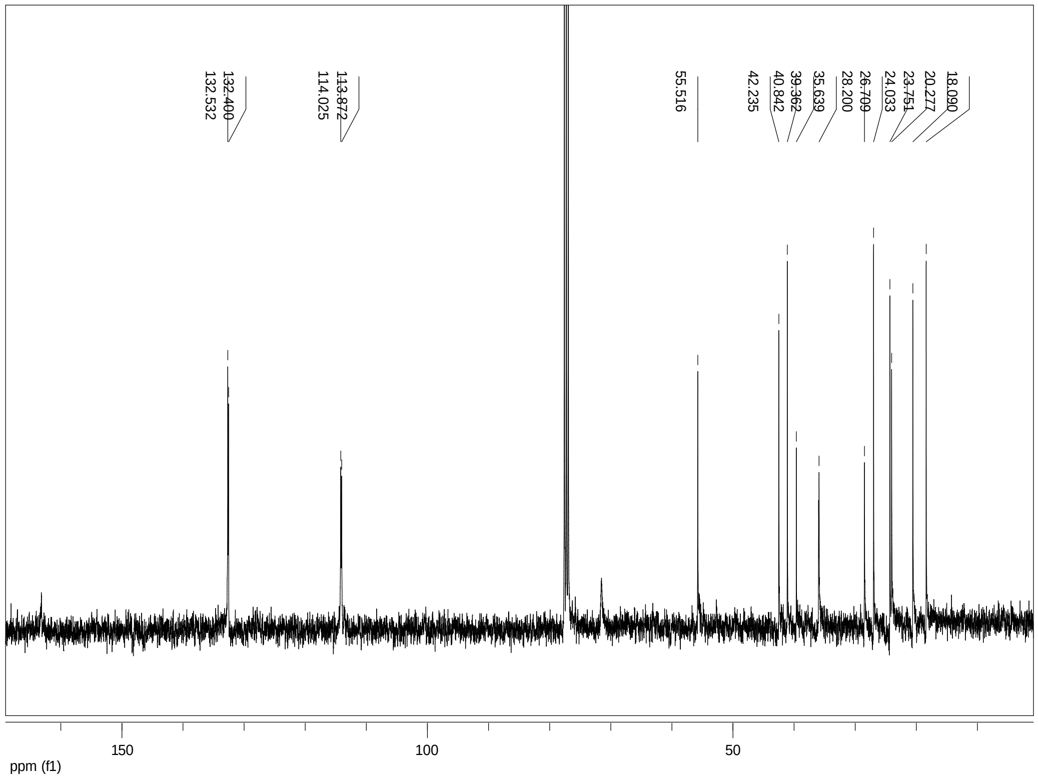

Fig. 23. ^{13}C-NMR spectra of the compound 1b

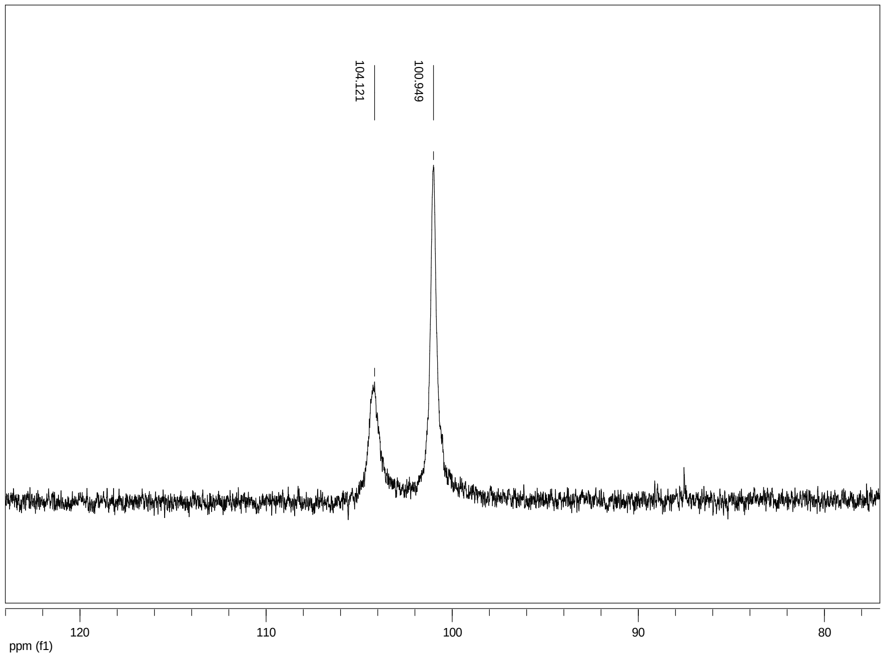

Fig. 24. ^{31}P-NMR spectra of the compound 1b

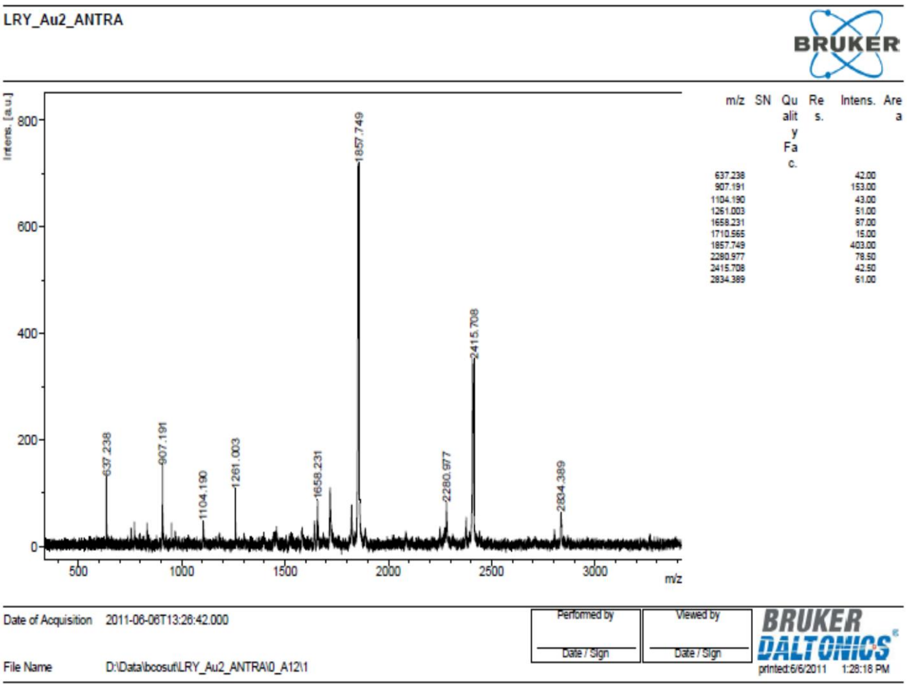

Fig. 25. Mass spectra of the compound 1b

7. [Ag{$R^1PS_2(OR^2)$}]$_2$ (R^1: 4-methoxyphenyl and R^2: (1S,2S,5S)-(-)-myrtanyl) 1c

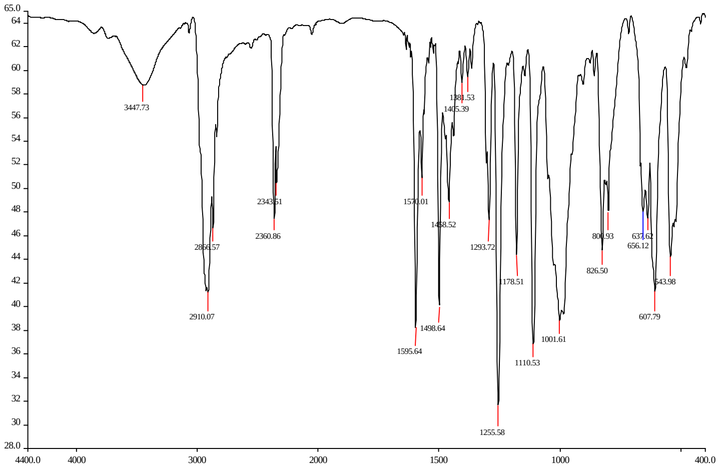

Fig. 26. IR spectra of the compound 1c

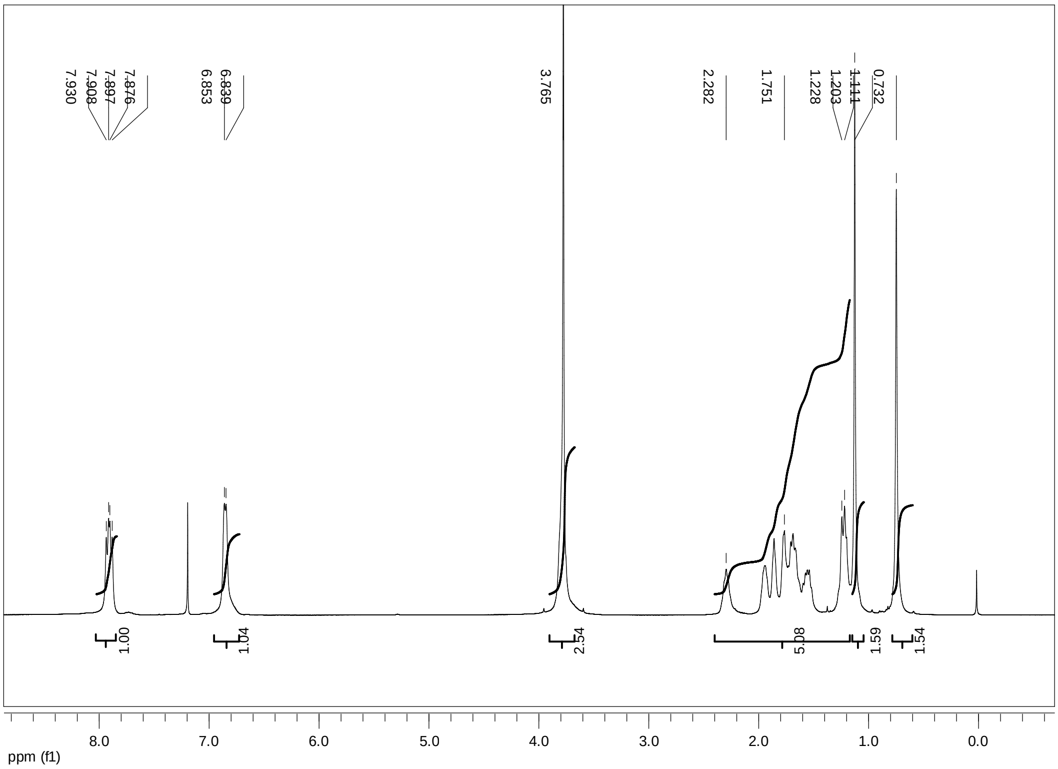

Fig. 27. ^{1}H-NMR spectra of the compound 1c

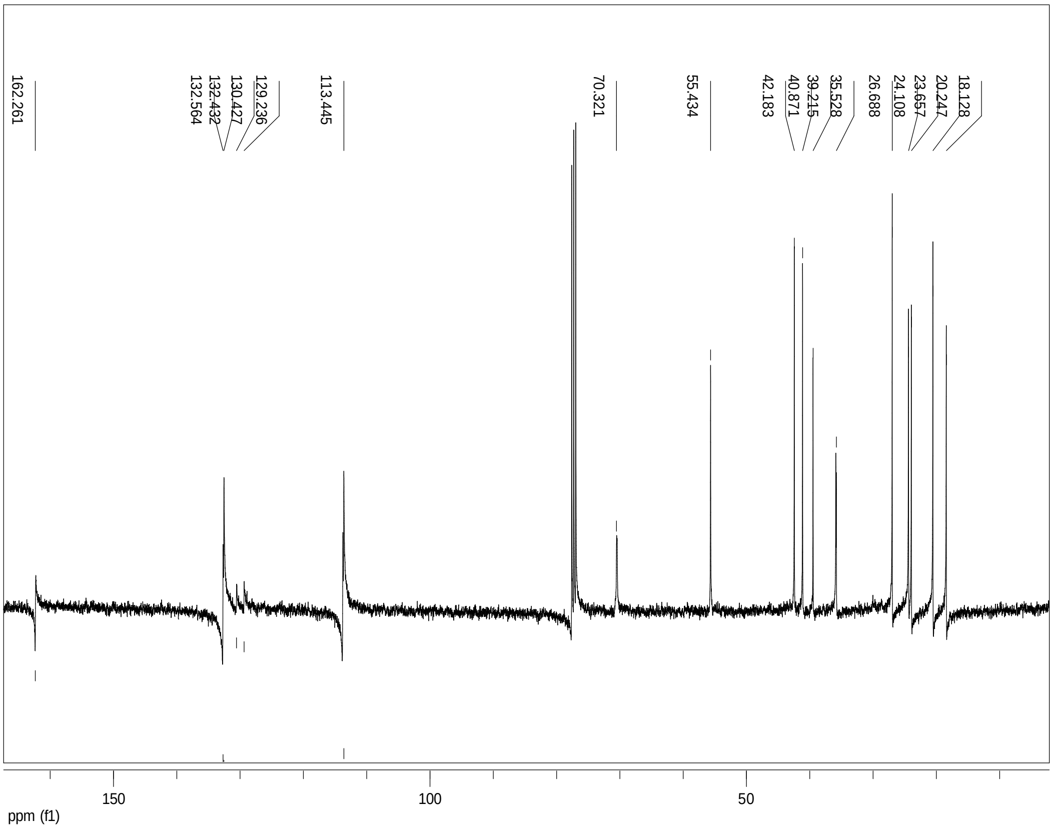

Fig. 28. ^{13}C-NMR spectra of the compound 1c

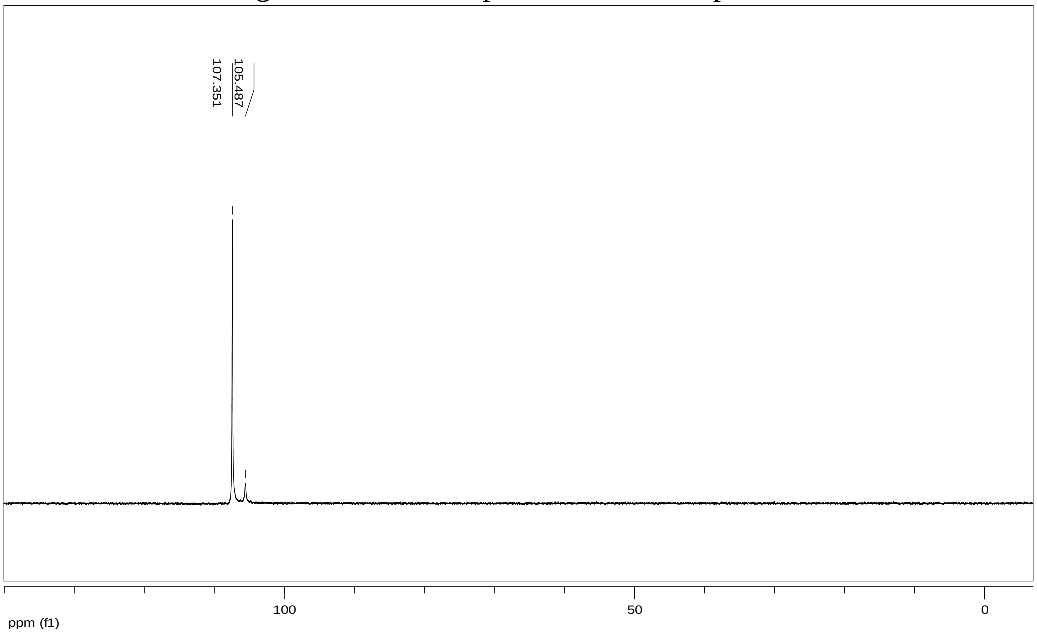

Fig. 29. ^{31}P-NMR spectra of the compound 1c

8. [Au(PPh$_3$)(R^{1}PS$_2$(OR^2)] (R^1: 4-methoxyphenyl, R^2: ((S)-(-)-O-(2-naphthyl)ethyl) 2a

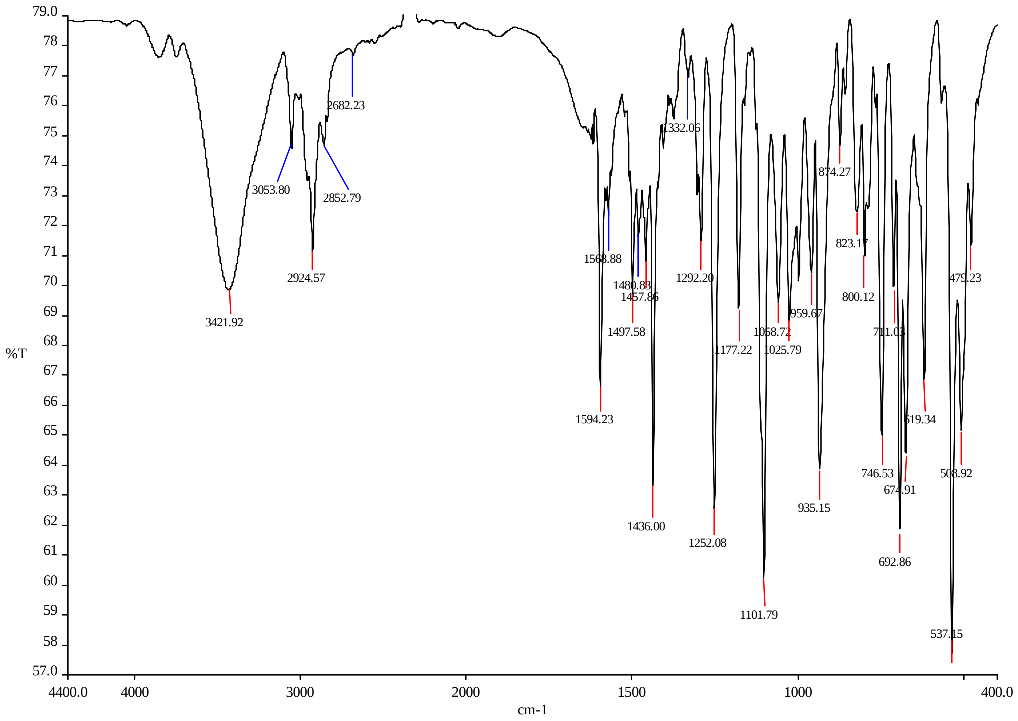

Fig. 30. IR spectra of the compound 2a

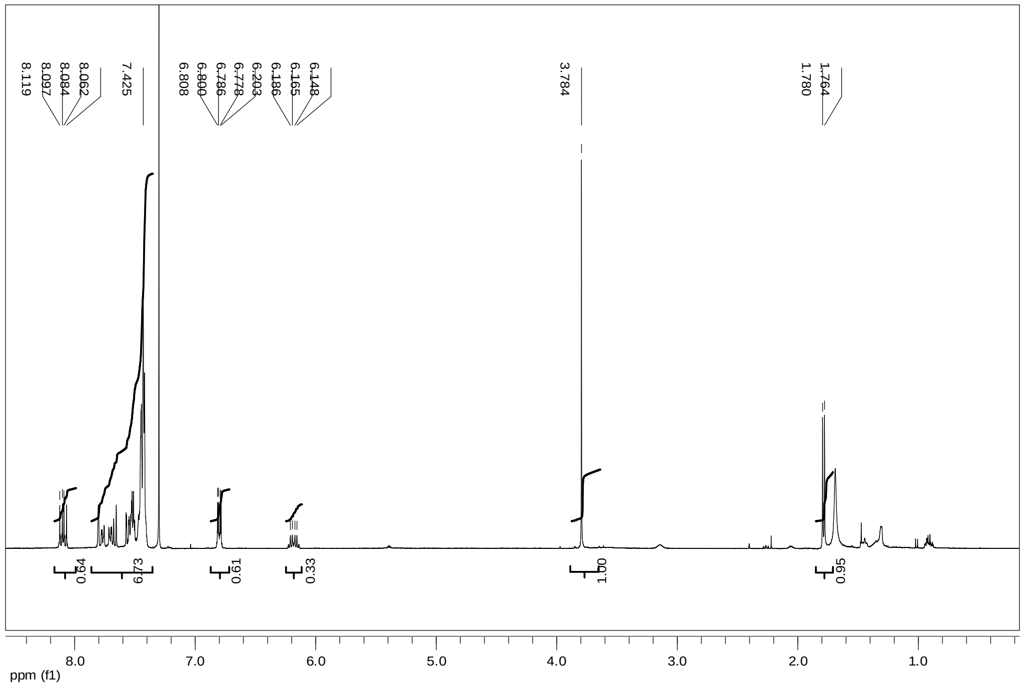

Fig. 31. ^{1}H-NMR spectra of the compound 2a

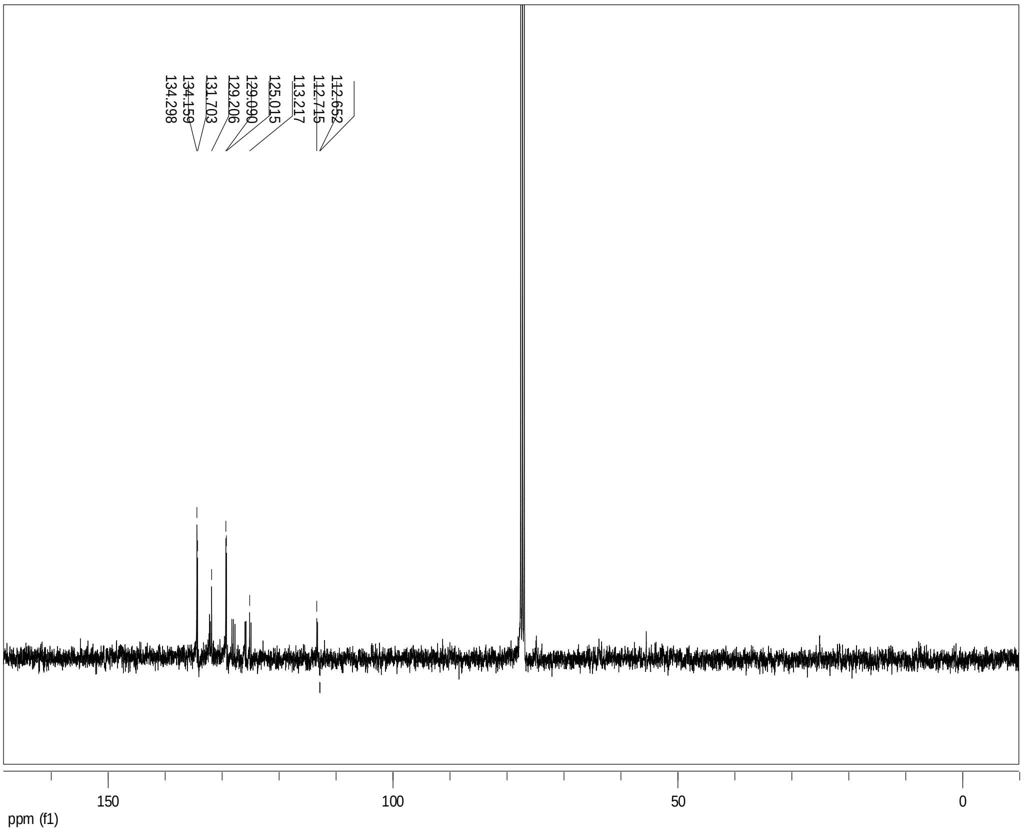

Fig. 32. ^{13}C-NMR spectra of the compound 2a

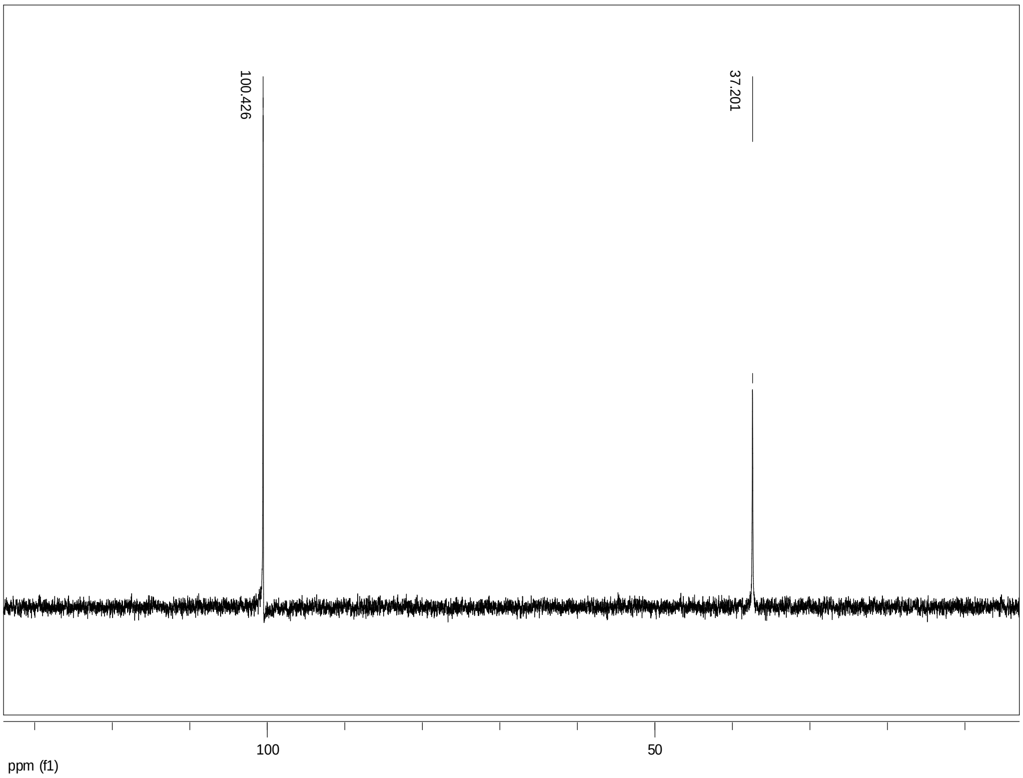

Fig. 33. ^{31}P-NMR spectra of the compound 2a

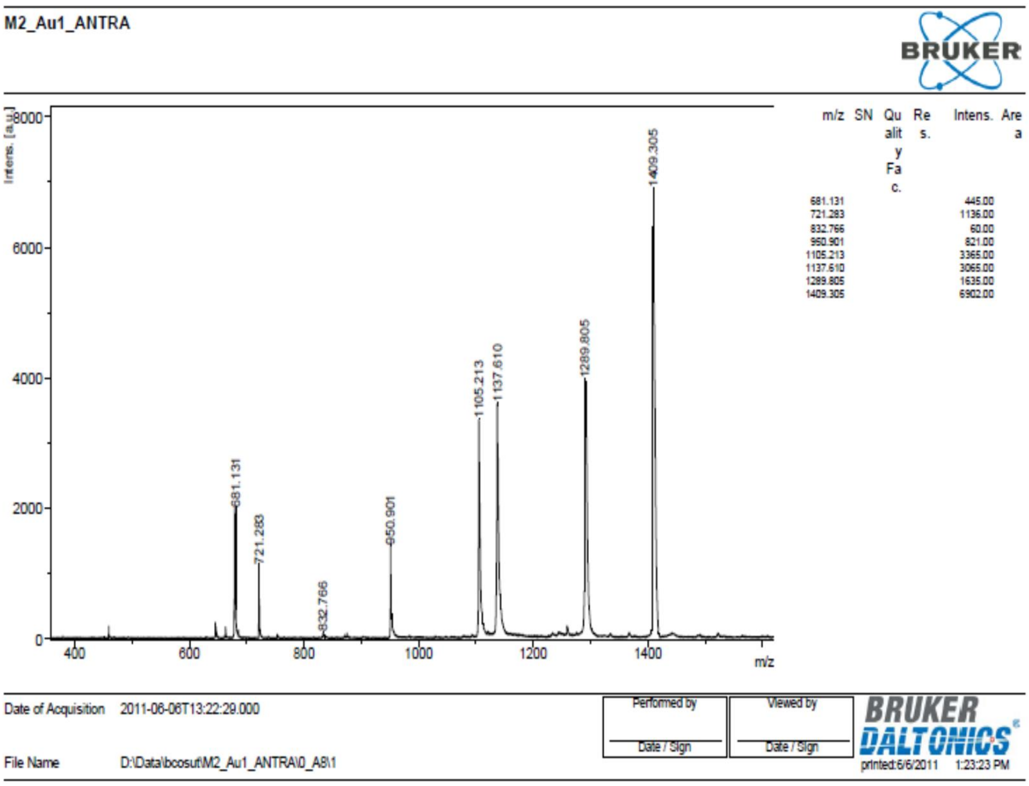

Fig. 34. Mass spectra of the compound 2a

9. $[Ag\{R^1PS_2(OR^2)\}]_2$ (R^1: 4-methoxyphenyl and R^2: (2-naphthyl)ethyl) 2b

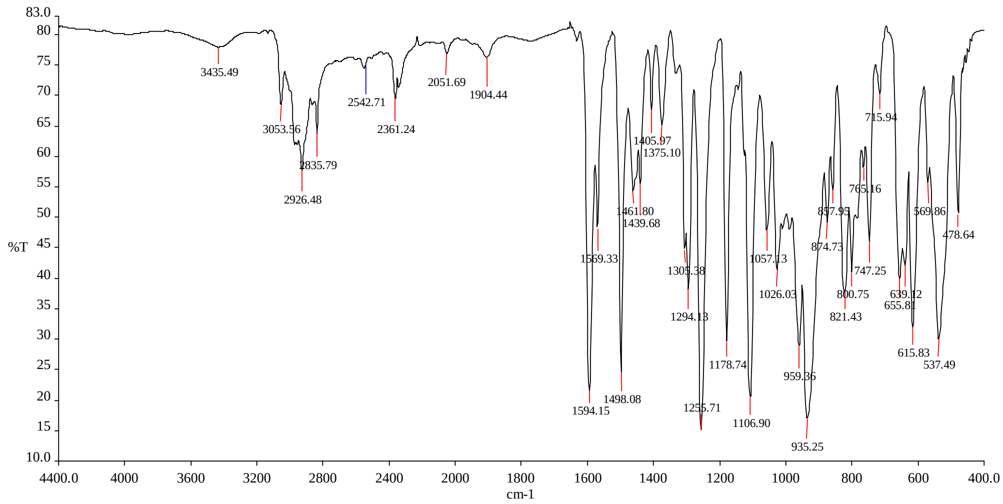

Fig. 35. IR spectra of the compound 2b

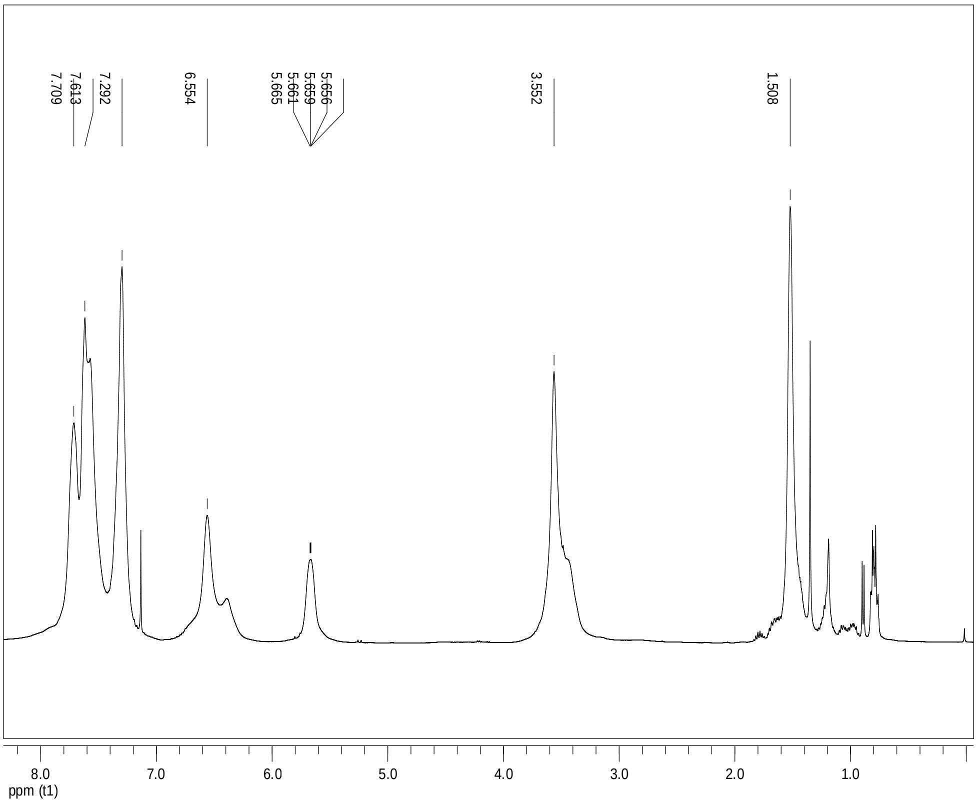

Fig. 36. ^{1}H-NMR spectra of the compound 2b

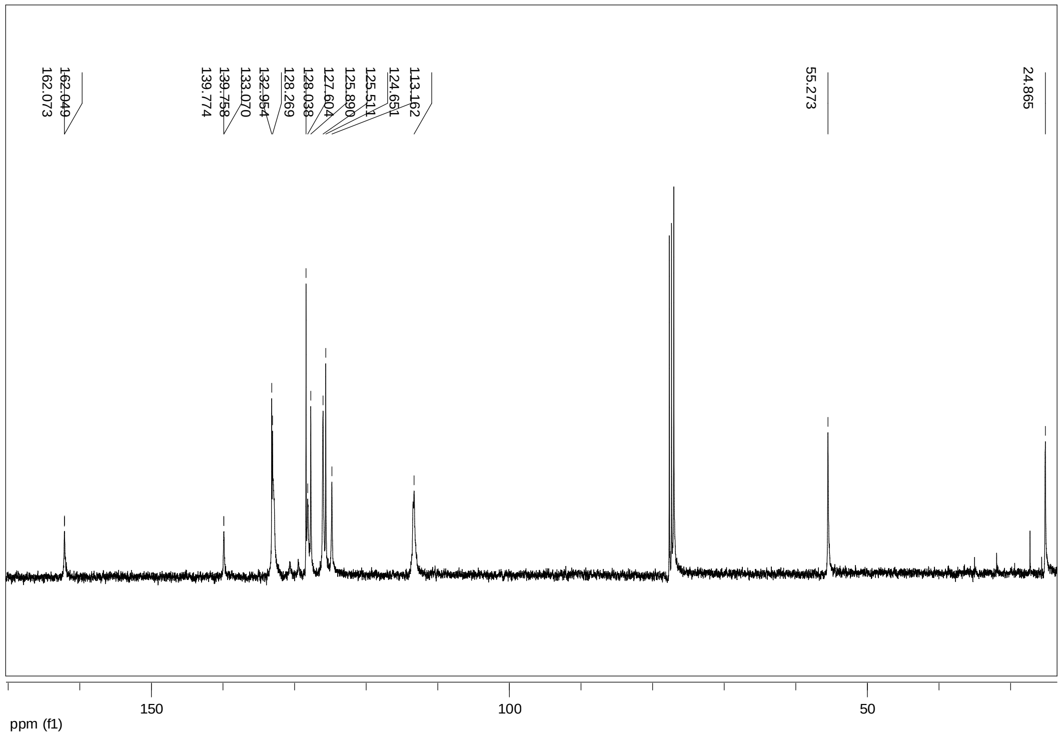

Fig. 37. ^{13}C-NMR spectra of the compound 2b

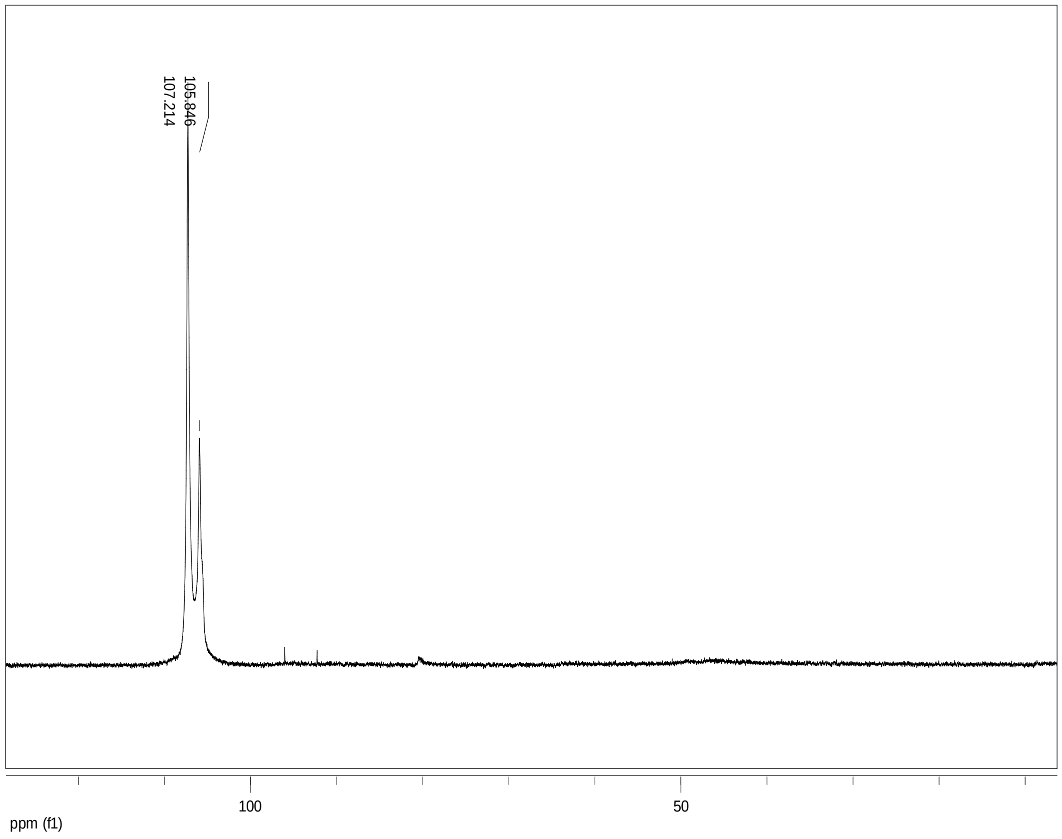

Fig. 38. ^{31}P-NMR spectra of the compound 2b

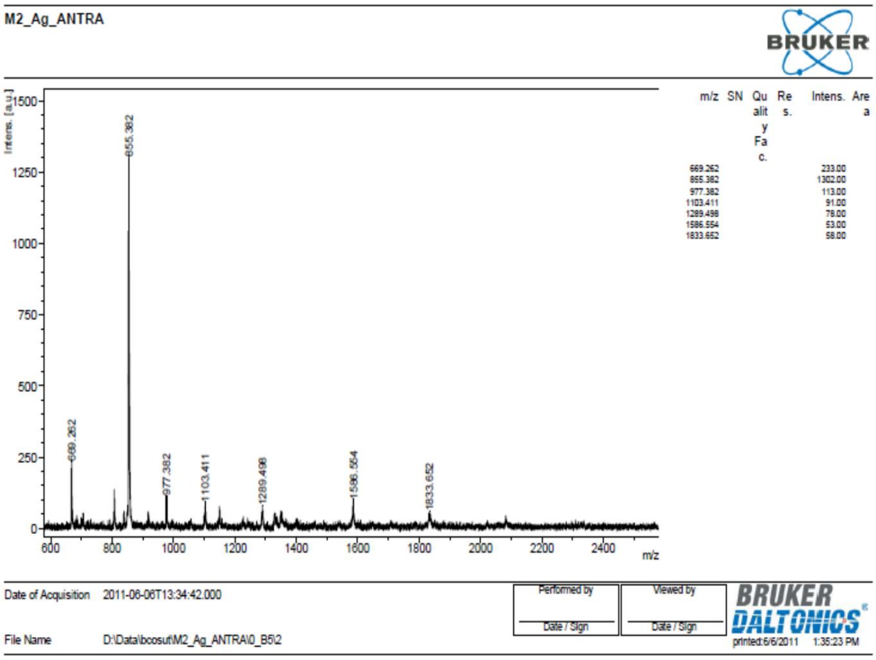

Fig. 39. Mass spectra of the compound 2b

10. [Au(PPh$_3$)(R^{1}PS$_2$(OR^2)] (R^1: 4-methoxyphenyl and R^2: (1R-(-)-myrtenyl) 3a

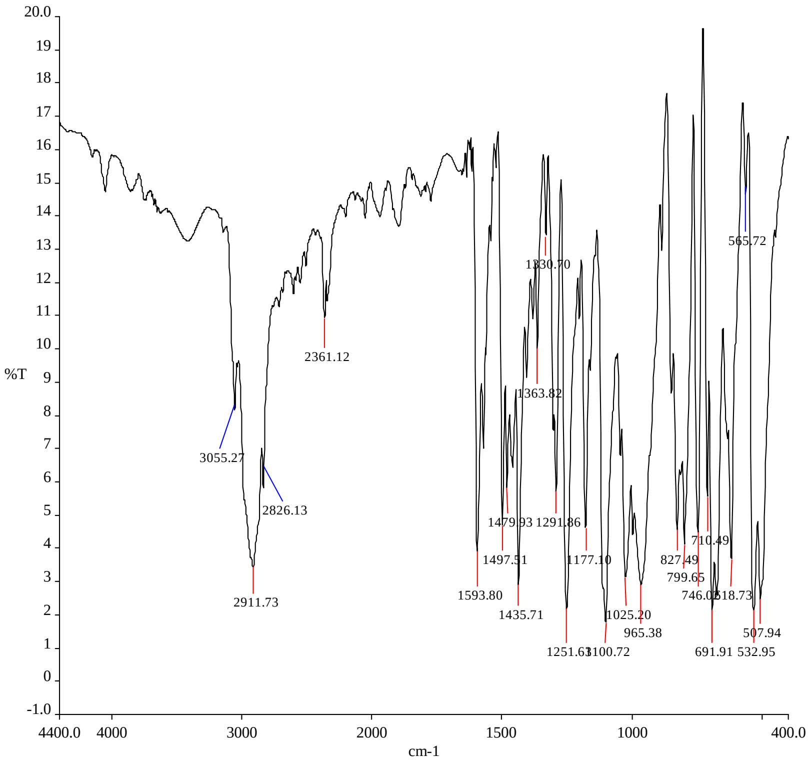

Fig. 40. IR spectra of the compound 3a

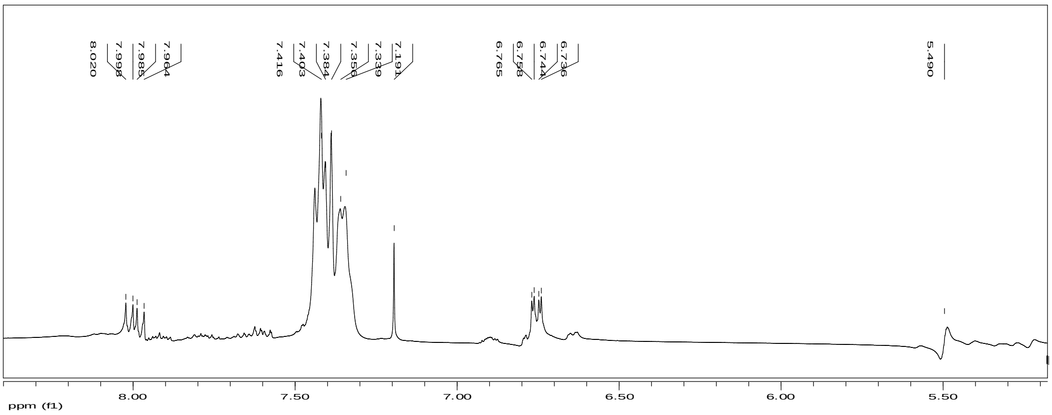

Fig.41a. ^{1}H-NMR spectra of the compound 3a

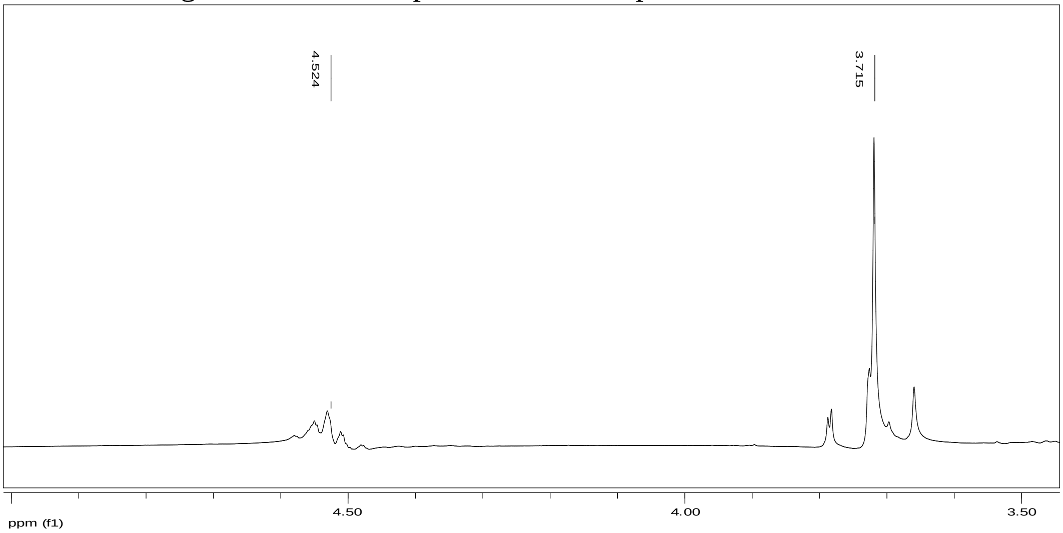

Fig. 41b. ^{1}H-NMR spectra of the compound 3a

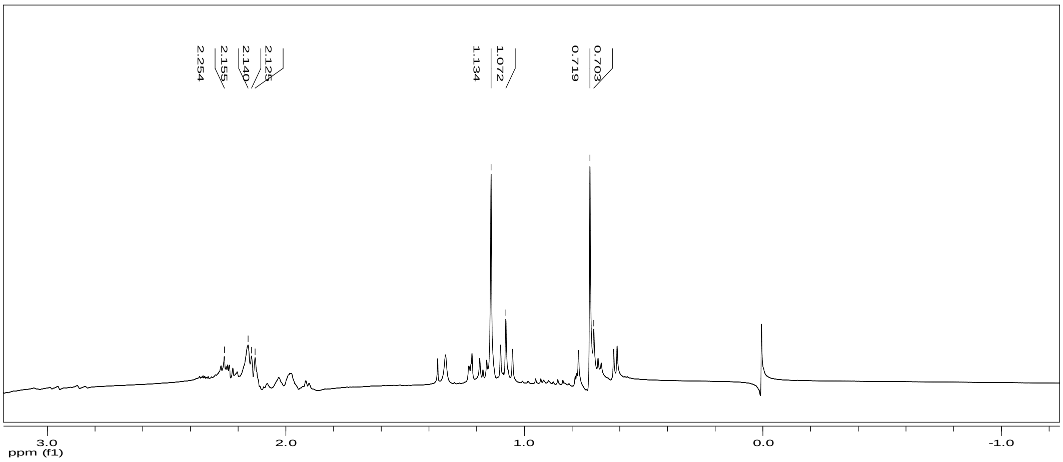

Fig. 31c. ^{1}H-NMR spectra of the compound 3a

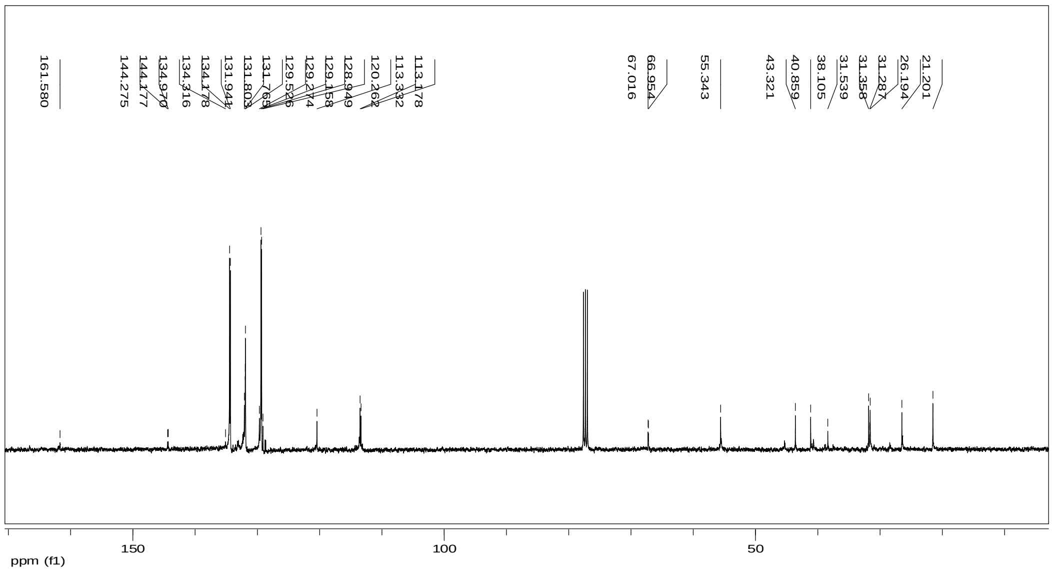

Fig. 42. ^{13}C-NMR spectra of the compound 3a

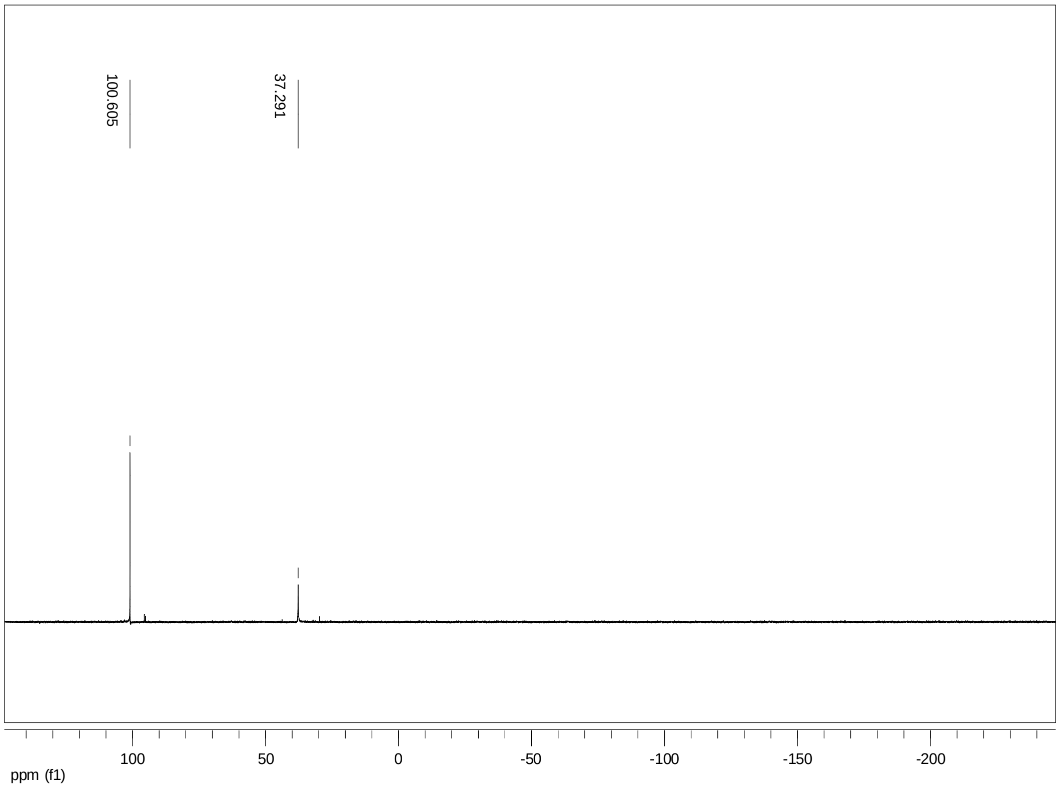

Fig. 43. ^{31}P-NMR spectra of the compound 3a

11. *Au_2 [O,O'-(Bornyl)$_2$PS$_2$] 4a*

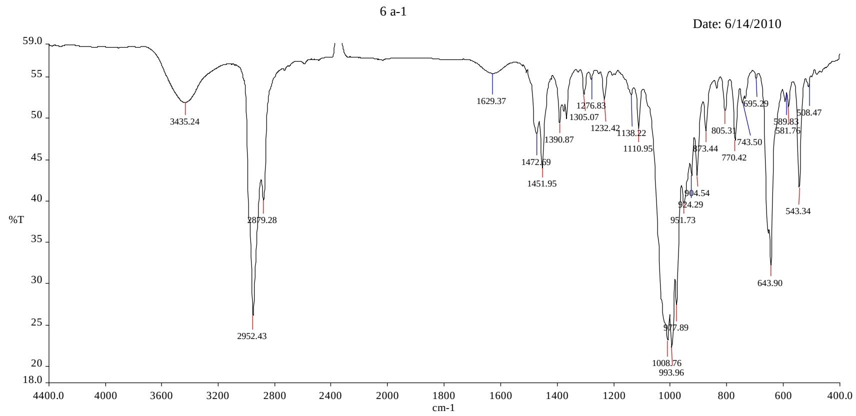

Fig. 44. IR spectra of the compound 4a

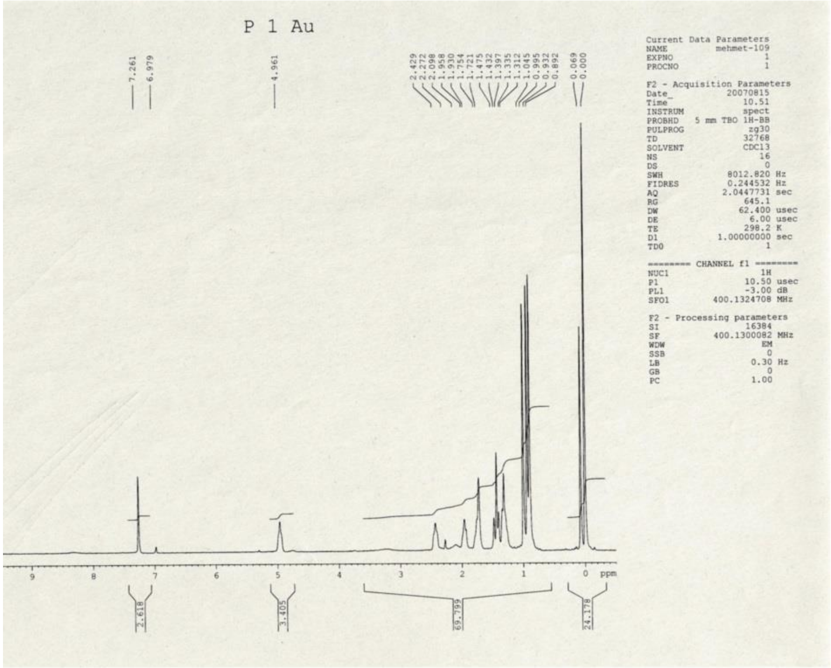

Fig. 44. ^{1}H-NMR spectra of the compound 4a

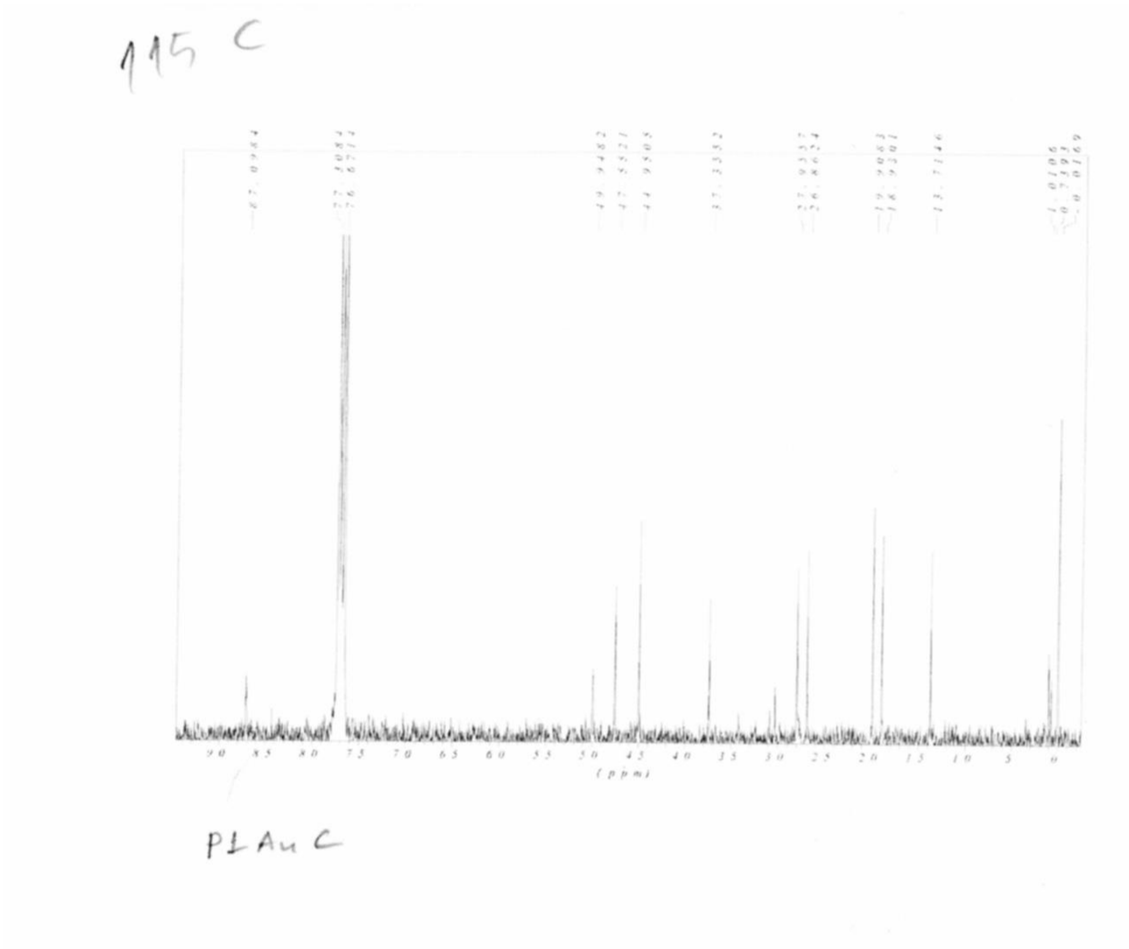

Fig. 45. ^{13}C-NMR spectra of the compound 4a

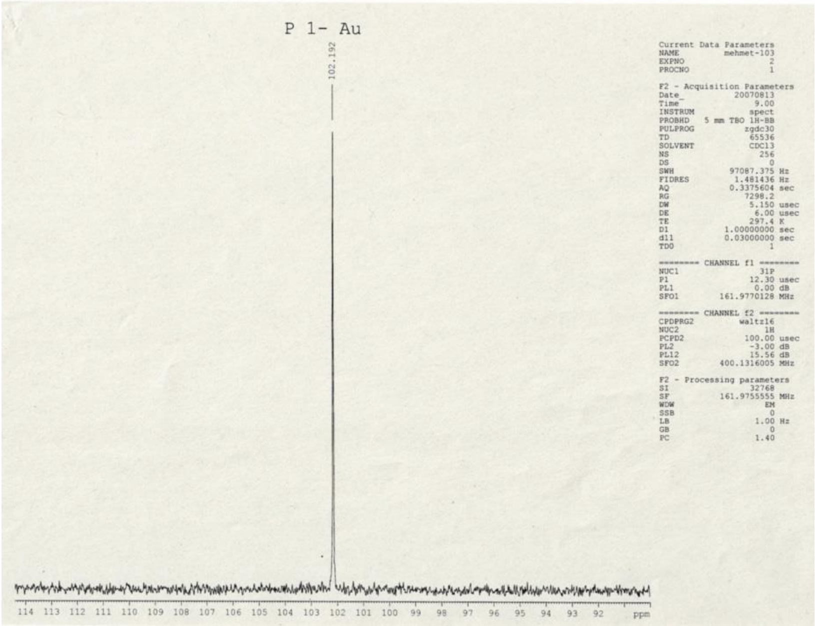

Fig. 46. ^{31}P-NMR spectra of the compound 4a
